# Spaceflight human optimization and performance: positioning astronauts in an operational framework to promote health, fitness, and mission readiness

**DOI:** 10.3389/fphys.2026.1950155

**Published:** 2026-09-21

**Authors:** Broderick L. Dickerson, Drew E. Gonzalez, Paul Comfort, John G. Hardy, Kelly E. Hines, Danielle N. Anderson, Timothy R. Macaulay, Aleksandra Stankovic, Corey A. Twine

**Affiliations:** 1 Exercise and Sport Nutrition Lab, Department of Kinesiology and Sport Management, Texas A&M University, College Station, TX, United States; 2 Occupational, Performance, and Nutrition Lab, Department of Kinesiology, Sam Houston State University, Huntsville, TX, United States; 3 Directorate of Psychology and Sport, University of Salford, Salford, United Kingdom; 4 Strength and Power Research Group, School of Medical and Health Science, Edith Cowan University, Joondalup, WA, Australia; 5 NASA Johnson Space Center, Houston, TX, United States; 6 Emergency Responder Human Performance Laboratory, Exercise and Nutrition Sciences, University at Buffalo, Buffalo, NY, United States; 7 CoachMePlus, Buffalo, NY, United States; 8 United States Air Force, Johnson Space Center, Houston, TX, United States; 9 Center for Space Medicine Research, Massachusetts General Hospital, Boston, MA, United States; 10 Department of Psychiatry, Harvard Medical School, Boston, MA, United States; 11 KBR Wyle Services, LLC., Johnson Space Center, Houston, TX, United States

**Keywords:** behavioral health, exercise countermeasures, long-duration spaceflight, operational readiness, space food systems

## Abstract

Optimizing astronaut mission readiness is a multidisciplinary endeavor that should integrate concepts from aerospace medicine, human performance, sports science, nutrition, and engineering. Situating astronauts within a theoretical framework similar to that of operational athletes, while addressing top human systems capability needs outlined for future missions, may be optimal for promoting sustained health and performance before, during, and after missions to low-Earth orbit, the lunar surface, and Mars. Mission readiness can be optimized by enhancing physiological and behavioral/psychological performance through data-driven and individualized exercise countermeasures, health and performance monitoring, precision nutrition, and rehabilitation strategies. Appropriate and stable space food systems that facilitate adequate provision of nutrients and fluids to prime astronauts for mission-critical tasks and operational demands are needed, though research is needed into potential nutritional considerations that might be useful for future missions. Behavioral readiness programs should emphasize stress inoculation, adaptive decision-making, communication, Earth-independent operation functionality, conflict resolution, leadership, and team-based problem-solving before flight to promote healthy team dynamics in-flight, along with proper society reintegration and uptake of family and occupational roles post-flight. Exercise remains an essential countermeasure during spaceflight, with an emphasis on maximizing fitness before launch to mitigate in-flight declines and on maintaining fitness levels and functionality during flight through high-intensity multimodal exercise. Following return to Earth, rehabilitative programs serve to restore cardiovascular, sensorimotor, and skeletal function, followed by enhancing skill-based performance components through a phase-based training program. Continuous monitoring systems that provide insight into health, performance, sleep, cognitive, nutritional, and recovery status should enable data-driven decision-making on physical readiness across all mission phases. By integrating insight from multiple disciplines, this unifying approach delivers a theoretical framework for sustaining astronaut health and performance during extended-duration missions.

## 1 Introduction

Long-duration spaceflight (LDS) imposes unique and novel stressors on human physiology and psychology in low Earth orbit (LEO), on or around the lunar surface, and eventually on Mars. Exposure to microgravity (μG), partial gravity, prolonged isolation and confinement, and increased radiation can cause multi-organ system deconditioning, affecting the musculoskeletal, cardiovascular, immune, and neurological systems (DeVirgiliis et al.; Turner and Robling, 2003; Comfort et al., 2021) (**Figure 1**). Future lunar-based and Mars-based (3–5 years) missions are going to exacerbate these risks and adaptations due to prolonged mission durations, decreased capacity for resupply, unique and altered space food systems, communication delays, increased need for autonomy, and unprecedented levels of radiation exposure, thereby providing new and unique challenges in maintaining crew health and function (Abercromby et al., 2025). Despite these challenges that threaten human health and mission success, crews must optimize and sustain health and performance during LDS.

**Figure 1:**
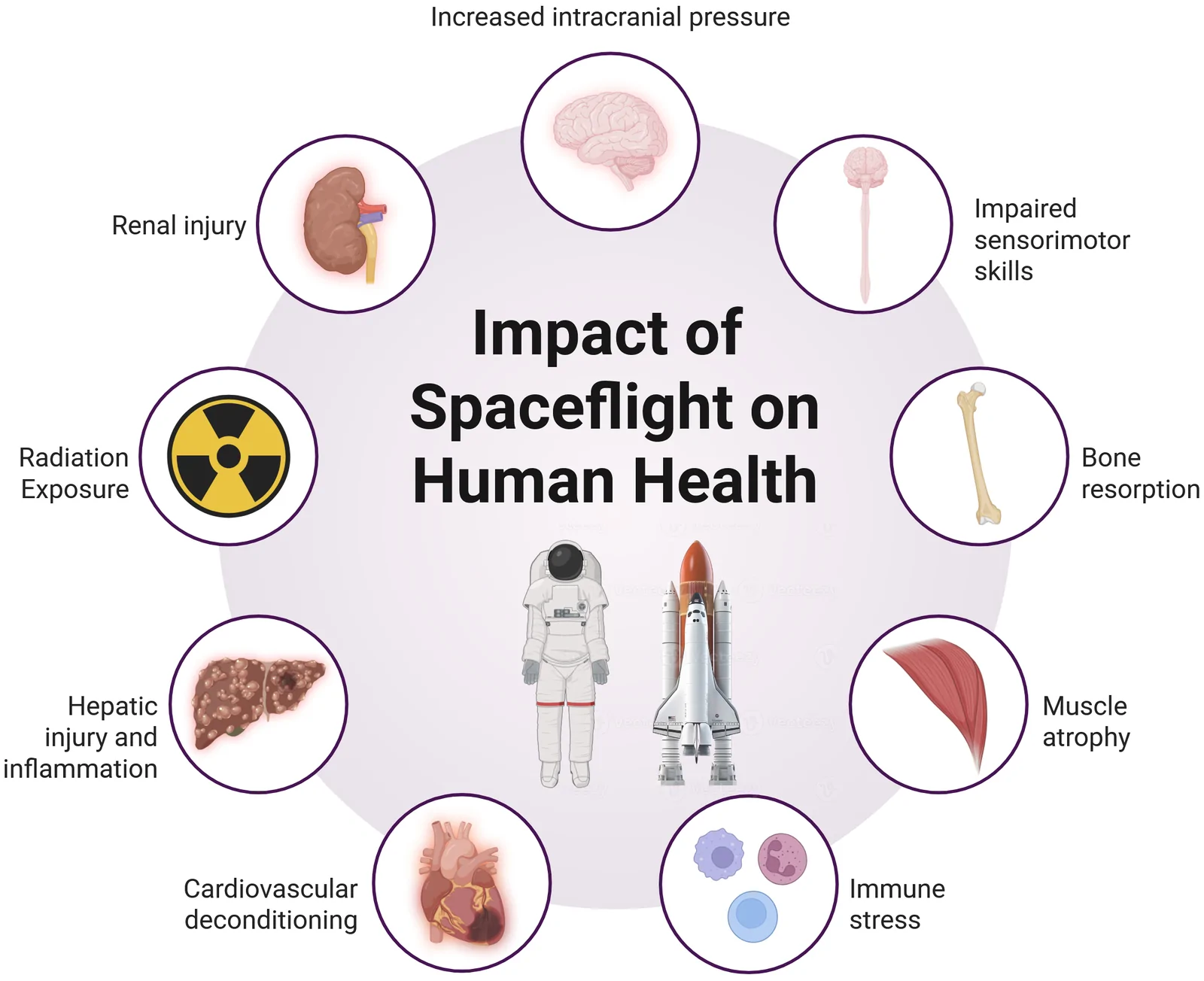
Impact of spaceflight on human health. Humans experience multi-system deconditioning during spaceflight with a number of bodily systems negatively impacted.

In recognition of these novel challenges, the National Aeronautics and Space Administration (NASA) recently identified eight critical human system capability needs for missions exploration-class missions: (1) Earth-independent human operations, (2) Mars-duration food systems, (3) risk mitigations in vehicular atmospheres, (4) computing models of injury and anthropometry, (5) exploration exercise countermeasures, (6) characterization of Mars-duration effects on human physiology, (7) sensorimotor countermeasures, and (8) strategies to address individual variation in responses to spaceflight (Varanoske et al., 2023; Abercromby et al., 2025). To meet these needs, a translational operational framework capable of monitoring, optimizing, maximizing, and maintaining astronaut health and performance across mission phases and durations is essential.

In this regard, sports science and human performance paradigms provide a plausible translational framework not only to address the challenges astronauts will experience in future missions but also to optimize their health and readiness before, during, and after spaceflight. Similar to athletes, astronauts are trained to meet the demands of specific activities and must sustain peak physical and cognitive performance under severe conditions, with little room for error. Athlete monitoring that is systematic and structured, involving fatigue and recovery evaluation (acute preparedness) and chronic adaptation (i.e., aerobic capacity, strength) status assessments, is imperative for leveraging workload alongside recovery and reducing the risk of injury and performance decrements (DeVirgiliis et al.). Overall, this model promotes individualized, multifaceted countermeasures and structured monitoring capabilities that optimize astronaut readiness for LDS, regardless of duration and mission phase (pre-, during-, and post-flight). Additionally, this integrative approach relies on collaboration with members of multiple disciplines and aims to apply concepts from evidence-based practices in athletic performance domains. This approach aligns health and mission status monitoring, evaluation, and countermeasure prescription strategies with the demands of novel LDS endeavors that will become longer and more complex than previous missions. Due to the multi-systemic alterations during LDS, considerations must be made pre-flight, in-flight, and post-flight that address and include exercise countermeasures, recovery strategies, space food nutrition, and monitoring of health status using technological devices (**Figure 2**).

**Figure 2:**
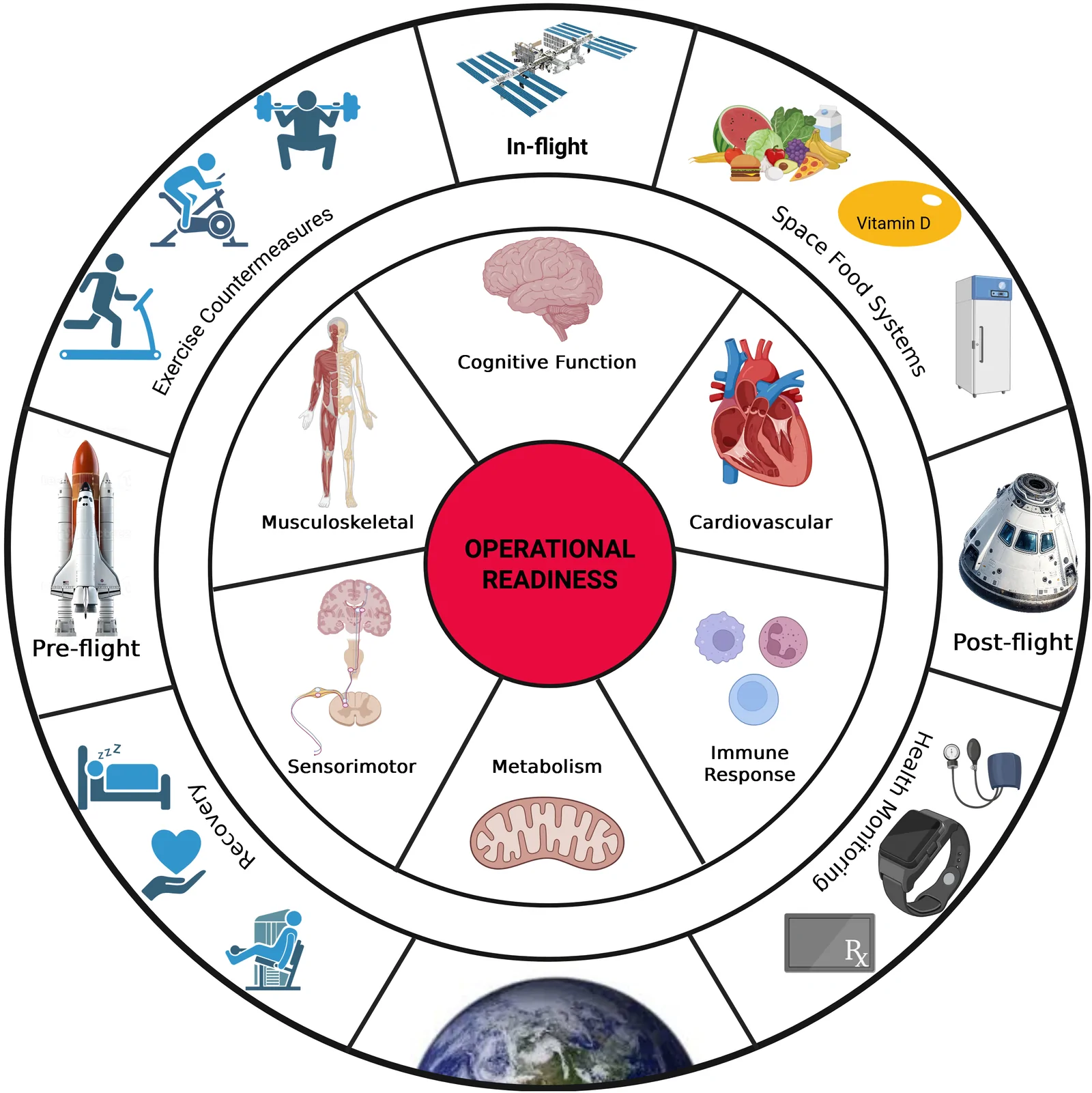
Operational readiness. Astronaut operational readiness is affected by systemic adaptation and the numerous countermeasures employed to maintain health and performance. Considerations during pre-flight preparedness, during-flight performance, and post-flight recovery should include exercise, nutrition, health monitoring, and recovery.

The authors of this narrative review propose an operational readiness framework that positions astronauts as operational athletes and couples NASA human-system capability needs with pragmatic monitoring strategies and multifaceted countermeasures that address nutritional, psychological, and physiological considerations.

## 2 Literature search methods

A narrative review with a systematic approach was used to collect eligible studies for this manuscript. A comprehensive search of four databases—PubMed, Google Scholar, ScienceDirect, and publicly available NASA research archives—was conducted between June 2025 and June 2026 to ensure the inclusion of possible recent findings. ResearchGate was used for full-text retrieval. Boolean operators included variations and combinations of “microgravity,” “spaceflight,” “astronaut,” “long-duration spaceflight,” “Mars,” “exploration-class missions,” “lunar,” “nutrition,” “space food systems,” “energy intake,” “macronutrients,” “micronutrients,” “exercise countermeasures,” ‘resistance training,” “aerobic training,” “power training,” “behavior,” “team dynamics,” “isolation and confinement,” “cognitive function,” “sleep restriction,” “health and performance monitoring,” “virtual reality,” “artificial intelligence,” “augmented reality,” “wearable devices,” “deconditioning,” “operational readiness,” and “sports science.” Peer-reviewed research and review articles, technical reports, human research program documents, and conference proceedings published in English were considered eligible for inclusion. No restriction on publication date was included as foundational physiological findings (e.g., from Skylab, Apollo missions) are presented. Given the narrative nature of this review, articles were selected by the author team based on relevance to the central domains discussed in the proposed operational readiness framework (nutrition, behavioral readiness, exercise countermeasures, rehabilitation and recovery, monitoring) and their applicability to current and future planned LDS missions.

## 3 Spaceflight food systems and nutrition

### 3.1 Space food systems

Operational space food systems are designed to meet the nutritional and energy demands of spaceflight while also adhering to mission constraints on mass, volume, shelf stability, waste management, and palatability. Novel space food/nutrition challenges are emerging as mission durations increase in complexity and length, with the planning of future lunar surface and Mars missions. Some apparent challenges include storage requirements, limited resupply capabilities, radiation exposure, communication delays, and increasing reliance on Earth-independent operations. Therefore, certain space food system considerations must be addressed to ensure operational readiness for astronauts (**Table 1**).

**Table 1:** Space food systems operational challenges.

Mission environment	Nutritional challenges	Operational implications
LEO	Limited menu fatigue, moderate storage requirements	Maintaining intake and dietary adherence
Lunar	Extended surface operations, intermittent resupply	Supporting EVA demands and recovery
Mars	Multi-year storage, no resupply, radiation exposure	Ensuring nutrient stability and Earth-independent food production

Historically, astronauts on the International Space Station (ISS) have relied primarily on shelf-stable, prepackaged foods that confer adequate macro- and micronutrients and are generally palatable among crewmembers (Douglas et al., 2016; Smith, 2021; Abercromby et al., 2025). Frequent supply of crew-preferred items (e.g., fruits, vegetables) has also historically been offered to mitigate menu fatigue and improve morale around meals. Although the current ISS space food systems support missions for at least one year (Kloeris, 2000), most menu items do not meet the shelf-life stability requirements needed for exploration-class missions (Douglas et al., 2021; Cooper et al., 2011; Perchonok and Douglas, 2018; Watkins et al., 2022). Therefore, continued optimization and exploration of alternative methods to enhance food stability, nutrient preservation, and crew acceptability are warranted, even for future LEO operations as these missions shift to commercial entities.

NASA’s current lunar space food strategy relies on shelf-stable, thermostabilized and irradiated pre-packaged menus for short-duration missions (e.g., Artemis II) while longer-duration surface operations involve development of *in-situ* production methods—including crop cultivation, 3D-printed food, and extended-shelf-life packaging—to reduce resupply (Smith et al., 2021). For future lunar missions that will focus on introducing a significant human presence on the lunar surface, additional constraints will be placed on the operating food systems while simultaneously increasing demands associated with surface operations and extravehicular activities (EVAs). Limited menu variety, prolonged reliance on shelf-stable foods, and reduced opportunities for fresh food resupply may adversely affect energy intake, nutritional status, crew morale (Smith et al., 2021), and could promote menu fatigue. Consequently, space food and nutritional strategies that promote dietary variety, thereby reducing potential menu fatigue and supporting adequate energy intake, will be essential during future Artemis missions.

Currently, no operating space food systems exist that would be acceptable for Mars missions. The nutritional demands of Mars missions are expected to exceed the capabilities of currently available food systems due to extreme mission durations and distances from Earth, which preclude resupply. Accordingly, long-duration exploration missions will likely require integrated food systems that combine advanced food preservation technologies with bioregenerative food production approaches to enhance nutrient stability, dietary variety, and Earth-independent operations. Technologies such as microwave-assisted thermal sterilization, high-pressure processing, and space-based horticultural systems have been explored and show promise (Jiang et al.; Zwart et al.; Catauro and Perchonok, 2012; Becker, 2013; Cooper et al., 2017; Escobar and Nabity, 2017; Munns and Nickelsen, 2017; Perchonok and Douglas, 2018; Helisch et al., 2019; Nangle et al., 2020; Soni et al., 2020; Douglas et al., 2021). However, additional research on these technologies is needed before integrating them into operating food systems.

### 3.2 Nutritional considerations for operational readiness

#### 3.2.1 Energy availability

The ability of astronauts to be operationally prepared for missions depends on the availability of nutritional energy. One hallmark finding from Apollo spaceflight research was the constant state of negative energy balance with astronauts only consuming around 64% of their recommended daily energy intake (Smith and Zwart, 2008a). This negative state of energy balance predisposes astronauts to injury risks and decrements in health (i.e., decreased bone mineral density (BMD)) and performance (Rambaut et al., 1975; Vorobyov et al., 1983; Lane et al., 1997; Smith et al., 2001, 2005b; Sibonga et al., 2007). However, due to the proper administration of exercise countermeasures, improved space food systems and food formulations, and an enhanced variety of menu options, astronauts have recently corrected energy balance issues of the past. (Bourdier et al.; Smith et al., 2005b, 2012b). With these concepts outlined, insufficient caloric and macronutrient intake might again become relevant during exploration-class missions, though this remains to be seen.

During exercise and times of stress, human intramuscular and neurological processes rely on carbohydrates for bioenergetics, which may influence emergency responsiveness. Recommended dietary allowances for carbohydrate intake during spaceflight are similar to Earth-based intakes (50-55% of daily caloric intake from carbohydrates), with some emphasis also placed on daily fiber consumption reaching ≥ 40g/d for gut and cardiovascular health (Smith, 2021) (**Table 2**). This is imperative because astronauts have historically not met adequate daily fiber intake during spaceflight (Smith, 2021). Coupled with inadequate total energy intake, impaired carbohydrate intake, plus inadequate fiber intake, may impair exercise performance, productivity, cognitive function, and the ability to perform mission-critical tasks (Sherman and Wimer, 1991; Gonzalez et al., 2022).

**Table 2:** Nutritional recommendations to optimize mission readiness.

Target	Recommendation	Rationale
Energy	Avoid chronic deficit	Preserve lean mass
Protein	1.3–1.6 g/kg/day	Muscle protein synthesis, multi-organ metabolism
Carbohydrates	50–55% kcal	Exercise performance, responsiveness
Fat	25–35% kcal	Energy density
Fiber	≥40 g/day	Gut health
Hydration	2.1–2.5 L/day minimum	Cardiovascular function, thermoregulation
Supplements	Creatine, omega-3s, probiotics (future research)	Potential countermeasures

In addition to carbohydrates, dietary fat is a macronutrient that may become increasingly important during exploration missions with mass and volume constraints. Currently, and similar to athletes, it is recommended that astronauts consume roughly 25-35% of their diet as dietary fats (Baba et al.; Kreider et al., 2010) (**Table 2**). However, unsaturated fatty acid sources should be emphasized as the main dietary sources, depending on stability. Current spaceflight diets may promote unfavorable changes in circulating blood lipids, increasing the risk of dyslipidemia among astronauts (Smith, 2021). Omega-3 fatty acids are of particular interest because they have been reported to support musculoskeletal, cardiovascular, cognitive, and immune health (Hac-Wydro and Wydro; Jäger et al.; Lunn and Theobald, 2006; Calder, 2015; Coniglio et al., 2023). However, the susceptibility of unsaturated fatty acids to oxidative degradation presents substantial challenges for long-duration missions (Martín‐Polvillo et al., 2004; Zhuang et al., 2022), namely in the wake of increased radiation exposure during Mars missions. Therefore, methods that aim to mitigate degradation and improve the stability of omega-3 fatty acids during LDS need further exploration (Du et al., 2022; Singh et al., 2022).

#### 3.2.2 Preservation of musculoskeletal function

Mission readiness is also contingent upon the health and integrity of skeletal muscle, which can be facilitated through adequate daily intake of protein, combined with resistance training. It has been empirically established that skeletal muscle atrophies during LDS, which dampens force production and power output. Maintaining adequate levels of protein intake is essential to combat these maladaptive processes. A common suggestion in the astronaut nutrition literature is that astronauts should consume approximately 1.3–1.6 g/kg/d to support anabolic processes and tissue protection (**Table 2**). Of note, essential amino acids (EAAs) should remain a target nutrient for astronauts as they govern muscle protein synthesis (MPS) and are integral nitrogen donors for synthesizing other amino acids (Dickerson et al., 2023). Furthermore, research suggests that μG dramatically influences amino acid metabolism, which highlights the importance of consuming all amino acids in the diet, not just EAAs (Garrett-Bakelman et al., 2019; Dickerson et al., 2023). For instance, conditionally essential amino acids become essential in catabolic conditions (i.e., μG) and can upregulate MPS, enhance immune response, and influence nitrogen balance (Ferrando et al., 1999; Wu, 2013; Wang et al., 2018; Viola et al., 2019; Smith et al., 2021; Dickerson et al., 2023).

Bone also deserves attention when formulating nutritional considerations for future lunar and Mars missions. Alongside resistance training and energy availability, micronutrients (i.e., vitamin D, calcium, boron, vitamin K, magnesium) provide health-conferring benefits on bone (Smith et al., 2005a, 2012a; Smith, 2021; Sibonga et al., 2024). In addition to adequate macronutrient intake, micronutrient delivery must be emphasized during LDS to prevent bone atrophy, among other benefits. Though current practices of astronauts do include supplementing vitamin D, low plasma concentrations have still been reported during spaceflight (Smith et al., 2005b), which calls for further nutrition-related interventions. Nevertheless, nutritional practices and systems that operate to preserve musculoskeletal integrity should remain a priority for future LDS missions.

#### 3.2.3 Cognitive, immune health, and metabolism support

Beyond muscle and bone, micronutrients are integral for cognition, immune response, and the body’s ability to use macronutrients for energy (i.e., bioenergetics). For instance, B vitamins are cofactors in bioenergetic pathways. Iron and zinc are important for the synthesis of neurotransmitters. Vitamins A, C, and E act as potent antioxidants, with C and E facilitating glutathione synthesis (Shenkin, 2006; Awuchi et al., 2020). The biological roles micronutrients play highlight the importance of their delivery during LDS. The stability of micronutrient storage during LDS remains an issue when planning exploration missions; however, reports suggest that astronauts typically consume amounts above recommended allowances during spaceflight (Smith et al., 2021).

It has already been established that carbohydrates and certain fatty acids are imperative for cognitive processes. However, micronutrients involved in neurological and metabolic function, such as vitamins B1, B2, B6, and D, as well as minerals involved in mitochondrial metabolism and blood glucose regulation, such as copper, iron, and chromium (Intakes et al., 2002; Aschner and Aschner, 2005; Feng et al., 2018) should receive considerable attention. Muscle and bone atrophy are mediated by mechanical-unloading-induced efflux of certain molecules (minerals, amino acids, etc.), which compromises micronutrient storage in tissues, thus potentially affecting functionality and cognition (Cooper et al., 2017; Field et al., 2022). In addition, astronauts are susceptible to insulin resistance during spaceflight due to insufficient mechanical stimulus and altered glucoregulatory metabolism (Intakes et al., 2002; Aschner and Aschner, 2005; Feng et al., 2018). Therefore, nutritional strategies to preserve metabolic health during LDS warrant further exploration.

An additional nutritional intervention target is the gut microbiome, which has links to the brain and skeletal muscle via neurotransmitters, chemokines, and/or short-chain fatty acids (Foster and Neufeld, 2013; Zhu et al., 2025). The microbial composition of the gut is extremely sensitive to μG radiation, dietary composition, isolation, and stress, all of which can impair gastrointestinal barrier function and alter immunity (Chen et al., 2016; Hao et al., 2018; Garrett-Bakelman et al., 2019; Dickerson et al., 2023). Therefore, probiotics and prebiotics may serve as novel nutritional and adjunctive countermeasures due to their reported ability to promote microbial diversity, support mucosal integrity, and attenuate inflammation (Mikelsaar and Mändar, 2021; Bharindwal et al., 2023). Introducing new bacteria (probiotics) in astronaut diets may promote favorable changes in gut composition, leading to health benefits. Also, increasing fiber (prebiotic) intake may represent a practical strategy for supporting gastrointestinal health during future LDS. However, research is needed to determine the appropriate administration of probiotics and prebiotics.

#### 3.2.4 Hydration

Hydration is another fundamental consideration for astronaut health in all spaceflight missions. Proper hydration strategies can influence cardiovascular function and hemodynamics, thermoregulation, and even cognitive function (Dickerson, 2025). Body water dynamics are influenced by μG, which causes a cephalad shift in fluids and transient decreases in plasma volume (Leach et al., 1996; Smith et al., 2021). These adaptations may impair cardiovascular function and contribute to orthostatic intolerance that is typically experienced upon return to Earth. Additionally, dehydration is a risk during flight due to operational demands and reportedly reduced sensation of thirst (Whitson et al., 1993, 2001; NASA, 2023). As a result, dehydration can increase renal stone risk and negatively impact capabilities essential for mission operations (thermoregulation, exercise and EVA capacity, cognition) (Scheuring et al., 2007; Etlin et al., 2024). These concerns become important during prolonged EVAs and lunar and/or Martian surface operations, where thermoregulation and energy expenditure are thought to increase dramatically (Etlin et al., 2024). Consequently, a space food system that includes appropriate delivery of fluids and electrolytes is essential. Current recommendations suggest that female astronauts consume 2.1 L/d and males, 2.5 L/d, with additional fluid, glucose, and electrolyte intake required during and following EVAs (Smith, 2021) (**Table 2**).

#### 3.2.5 Alternative nutrition strategies and precision monitoring

Lunar and Mars missions will introduce unprecedented levels of challenges regarding nutritional requirements in astronauts. Future research should examine alternative, operable food systems that promote food stability and palatability while identifying nutritional countermeasures that may enhance operational readiness. Historically, supplementation with diets, ingredients (aside from vitamin D), ergogenic aids, etc., has been dismissed in spaceflight nutrition. However, reexamination of the potential need for supplementation, depending on stability and delivery logistics, is warranted due to the currently unknown challenges that astronaut nutrition will face in future LDS.

One ergogenic, health-promoting nutrient that has received marked attention in human performance and clinical research is creatine monohydrate (CM). Evidence from numerous trials have exhibited the safety of CM supplementation and positive effects on skeletal muscle, exercise performance, immune response, neurological health, cognitive function, organ radioprotection, and longevity, yet it has not fully been evaluated in spaceflight (Ahmun et al., 2005; Candow and Chilibeck, 2007; Rawson and Venezia, 2011; Ellery et al., 2016; Dean et al., 2017; Amirsasan et al., 2018; Avgerinos et al., 2018; Antonio et al., 2021; Saito et al., 2022; Nersesova et al., 2024; Xu et al., 2024; Kreider et al., 2025). Although some evidence does suggest in Earth-based analogs that creatine may help maintain muscle mass and enhance recovery in post-operative and/or limb-unloaded conditions (Hespel et al., 2001; Aoki et al., 2004; Pakulak et al., 2022; Burke et al., 2023). Given its favorable safety profile and pleiotropic benefits, creatine represents a promising countermeasure for future missions, though studies are needed in its utilitarian implementation due to questions that remain in long-term stability, namely for Mars missions. Dry CM powder has shown to be stable for more than three years, even in elevated thermal environments (Jäger, 2003; Kreider et al., 2022); however, it is currently unknown what prolonged radiation exposure does to CM, either terrestrially or during spaceflight. Therefore, studies are needed on the ergogenic properties and the stabilization of CM exposed to radiation levels comparable to what is expected during Mars missions.

Lastly, precision nutrition strategies, combined with advanced monitoring technologies, will likely be implemented on future exploration missions to optimize astronaut readiness. Point-of-care biochemical analysis that enables continuous dietary status, wearable devices, and artificial intelligence (AI) may allow astronauts to implement individualized nutritional regimens based on physiological status, operational demands, and readiness metrics (Smith and Zwart, 2008b; Smith, 2021). Recently, researchers have explored such technologies in health monitoring for astronaut health status evaluation in LEO (Crucian et al., 2021; Axiom Space, 2025). However, more research is needed before appropriate implementation. Collectively, these technologies will allow astronauts to follow nutritional habits that can effectively optimize mission readiness.

### 3.3 SHOP perspective

Within the Spaceflight Human Optimization and Performance (SHOP) framework, nutrition can be used as a tool in space food systems to address mission demands. Generally, astronauts should consume 50-55% of daily caloric intake as carbohydrates, 25-35% as dietary fats, with 1.3 – 1.6 g·kg^−1^·day^−1^ of protein throughout the mission. Individualized nutrition strategies and sustainable food systems adequate for LDS for all future missions should be viewed as countermeasures that promote adequate energy intake and support for musculoskeletal health, cognitive function, recovery from exercise and EVAs, and operational performance in all phases of spaceflight. In-flight monitoring tools that assess nutrition and health status via biomarkers are also suggested to optimize mission readiness.

## 4 Behavioral health, cognitive performance, and operational readiness in exploration space missions

As missions extend from LEO to Mars, not only do durations increase but gravitational vectors change, radiation exposure increases, supply of materials diminishes, exercise countermeasure capabilities are reduced, and commination becomes progressively difficult (**Figure 3**). Direct spaceflight, Antarctic ground-based analogs, along with polar expeditions and other isolated, confined, extreme (ICE) analogs have consistently shown these environmental settings influence cognitive function, mood, team-based dynamics, and operational performance (Sandal et al., 2006; Kanas and Manzey, 2008; Palinkas and Suedfeld, 2008; Basner et al., 2014; Kanas, 2015; Pagel and Choukèr, 2016). Consequently, these adaptations can lead to catastrophic mission failures, thus situating behavioral health and cognition as mission-critical capabilities rather than secondary metrics.

**Figure 3:**
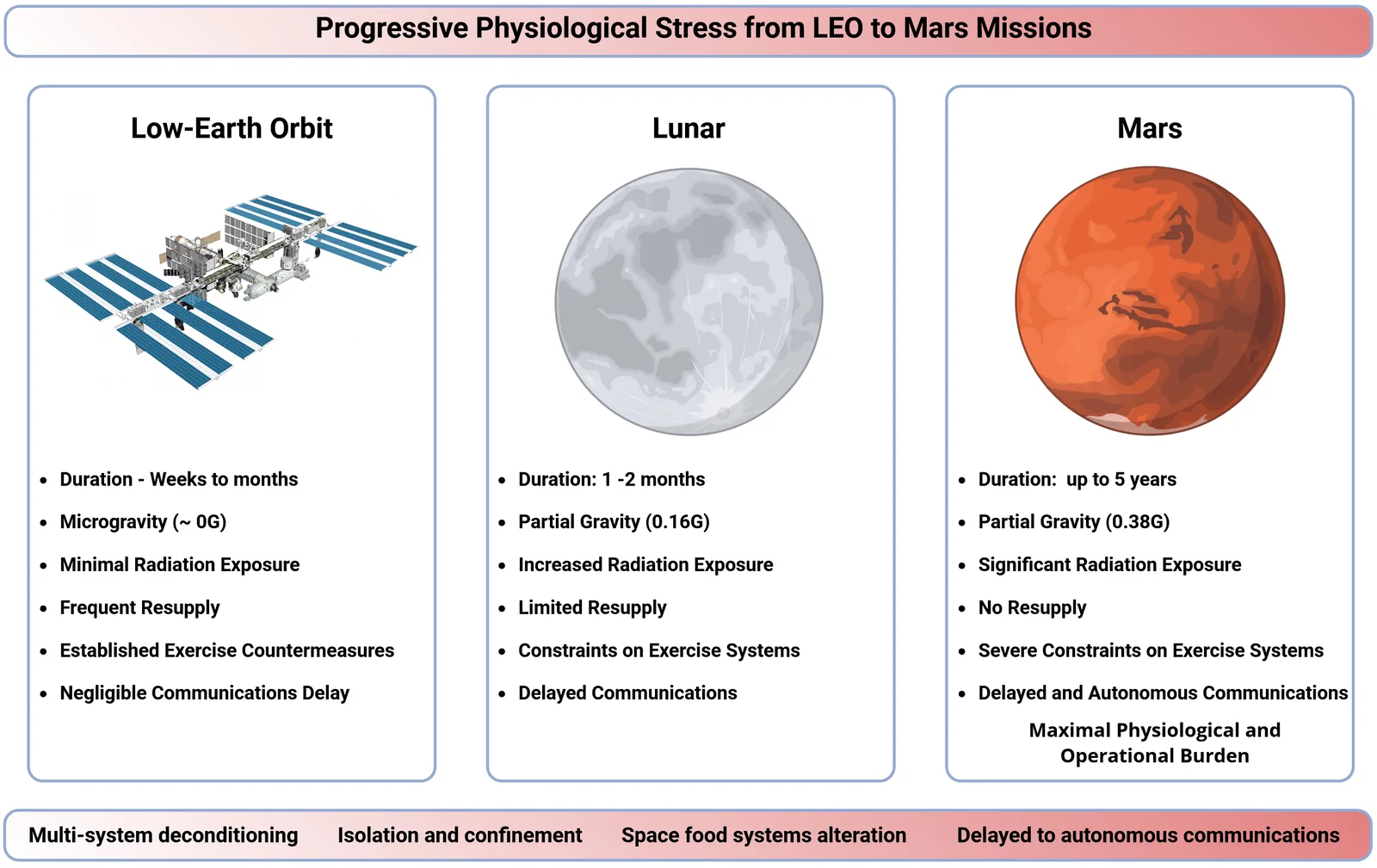
Progressive physiological stress from LEO to mars missions. As missions increase in duration and distance traveled from Earth, new physiological, behavioral, and technological challenges will arise.

As crews travel farther from Earth in Mars missions, this perspective is particularly important because traditionally utilized models of mission control consultation will become progressively less available. Accordingly, among the Earth-independent operations NASA has identified in human system capability needs, cognitive countermeasures, teamwork, and characterization of individual variability are key for lunar and Mars exploration (Abercromby et al., 2025). Within the collaborative operational readiness framework, behavioral preparedness should be trained during preflight preparation, involve in-flight monitoring and support, and be integrated into post-mission rehabilitation to ensure that crews can maintain high levels of performance (Sipes and Vander Ark, 2005; Kanas, 2015; Landon et al., 2015; Slack et al., 2016; Abercromby et al., 2025; Vakoch, 2011).

### 4.1 Behavioral and cognitive stressors in LDS

#### 4.1.1 Individual-level stressors

Behavioral stressors during LDS should be noted as dynamic and interactive issues that challenge intra- and interpersonal dynamics (Sandal et al., 2006; Basner et al., 2014; Pagel and Choukèr, 2016; Slack et al., 2016). Multiple physiological and psychological stressors such as sleep deprivation, circadian disruption, isolation and confinement, habitat fatigue, neurovestibular adaptation, and high operational tempo collectively influence behavioral health and cognition during LDS. Among these, sleep loss and circadian disruption remain the most well-established threats to astronaut performance. It is common for astronauts to experience insufficient sleep before, during, and after spaceflight. Research shows that sleep deprivation is associated with reduced psychomotor vigilance, impaired reaction time, and greater perceived workload and stress (Barger et al., 2014; Jones et al., 2022). Additionally, ground-based analog studies report that inadequate sleep impairs attention, executive function, working memory, and decision-making, and even anabolic responses, and exercise recovery which are capacities essential for emergency responsiveness and execution of complex procedures (Saner et al., 2020; Jones et al., 2022; Piechowski et al., 2024).

Opportunities for evacuation, resupply, and family contact diminish as mission durations increase along with exposure to ICE in future LDS missions. Evidence from Antarctic stations, Mars analogs, and other ICE environment studies has documented risks related to mood disturbance, reduced motivation, social withdrawal, increased interpersonal conflict, and altered time perception (Sandal et al., 2006; Palinkas and Suedfeld, 2008; Pagel and Choukèr, 2016). According to results from Mars-500 and other analogs, such behavioral effects may develop gradually and unevenly among crew members, with some even exhibiting relatively stable moods. Conversely, others experience increased stress, altered time perception, and/or reduced sociality (Basner et al., 2014). These adaptations may be further exacerbated by environmental monotony, which can reduce sensory stimulation and opportunities for psychological support (Lyons et al., 2020; Thoolen et al., 2025; White et al., 2025). Antarctic evidence of neurological and behavioral alterations further supports the possibility that extended ICE environments may influence cognition through both psychosocial and neurobiological pathways (Stahn et al., 2019).

Unique altered gravitational environments will also be introduced during future LDS missions. Neurovestibular and sensorimotor adaptations associated with G-transitions influence spatial orientation, balance, locomotion, manual control, and situational awareness (Bloomberg et al., 2015; Macaulay et al., 2021). During long-duration transit followed by landing on the moon or Mars, crewmembers may need to perform surface egress, mobility tasks, and early EVA operations while still adapting to a changed gravitational environment. The resulting decrements could interact with fatigue and workload to raise error risk and work inefficiency at the very moment crews must function autonomously and decisively (Bloomberg et al., 2015; Macaulay et al., 2021). For exploration missions, sensorimotor countermeasures will be required to ensure operational readiness (National Aeronautics and Space Administration, 2010; Macaulay et al., 2021; Abercromby et al., 2025).

#### 4.1.2 Team-level stressors

Teams that operate in ICE environments for extended durations must maintain healthy interpersonal dynamics via effective communication, trust, and cohesion in times of privacy limitations and operational strain. Team science research has consistently identified key determinants of effective team dynamics, including leadership, adaptability, mutual performance monitoring, backup behavior, and team orientation (Salas et al., 2005; Harris et al., 2024). During LDS, these actions influence team morale and mission operational safety due to compromised coordination and/or unresolved conflict among members, which can impair information sharing, reduce cross-checking, and increase the likelihood of trivial errors that lead to mission-threatening threats (Sipes and Vander Ark, 2005). Thus, maintaining effective team dynamics and healthy interpersonal relationships in the workplace should be emphasized as core characteristics of behavioral readiness training.

Exploration-class missions will require the implementation of increased autonomy, with more autonomous processes being utilized the further crews travel from Earth. This will place additional constraints on team demands. Communication delays during Mars missions may approach 20 min each way, eliminating the possibility of rapid consultation with mission control during time-critical situations. Under these conditions, and with limited guidance from ground control, crews must implement problem-solving tactics, share situational awareness, and detect anomalies (Landon et al., 2015; Abercromby et al., 2025). Team science and direct spaceflight research suggest that communication latency increases the importance of effective communication, adaptive and fluid leadership responsibilities, and cohesive mental models (Cannon-Bowers and Salas, 1993; Helmreich and Merritt, 1998; Landon et al., 2018; Verhoeven et al., 2022). Therefore, leadership roles should be shared/distributed with authority shifting based on expertise, action plans, and operational demands, compared to traditional missions (Helmreich and Merritt, 1998; Salas et al., 2005; Landon et al., 2018; Verhoeven et al., 2022). Multi-team collaboration and integration are also essential for exploration-class missions. Isolated crew activities are unacceptable for exploration missions, which are contingent upon the synchronization of multidisciplinary personnel within mission control, medical crew, and engineering specialists in times of uncertainty and during operational autonomy (Landon et al., 2018; Verhoeven et al., 2022).

### 4.2 Integrative effects on higher-order cognitive function

The stressors described above eventually influence higher-order cognitive processes that enable the successful completion of mission-critical tasks. Subdomains of executive function (e.g., working memory, attention, vigilance, and situational awareness) support problem-solving, issue resolution, and adaptive decision-making under uncertainty (Endsley, 1995; Wickens, 2008; Miyake and Friedman, 2012; Diamond, 2013). Characteristics of prolonged excessive workload, such as distress and fatigue, can impair components of executive function by affecting prefrontal cortical systems, resulting in acute and chronic cognitive impairment (McEwen and Sapolsky, 1995; Arnsten, 2009; Goel et al., 2009; Lim and Dinges, 2010). Additionally, cognitive biases may underpin human decision-making and may become amplified under uncertain, stressful conditions (McEwen and Sapolsky, 1995; Arnsten, 2009; Starcke and Brand, 2012). Accordingly, challenges to behavioral health should be considered as threats to well-being and contribute to mission-relevant changes in judgment, risk assessment, and problem-solving capabilities. From an operational perspective, maintaining cognitive function and behavioral readiness is essential, as even subtle decrements in executive function can compromise safety and decision quality, thereby threatening mission success during autonomous LDS. In-flight monitoring and countermeasures must therefore be designed for instantaneous performance, decision-making, and cognitive/behavioral support rather than retrospective diagnosis (Tversky and Kahneman, 1974; Endsley, 1995; Bechara et al., 2000; Arnsten, 2009; Starcke and Brand, 2012).

### 4.3 Behavioral readiness monitoring

Even though it has long been understood that behavioral factors influence mission success, current monitoring strategies remain limited in logistical validity, temporal resolution, and integration with real-time decision-making during operational tasks. Traditional approaches, such as self-report questionnaires, behavioral meetings, sleep logs, and periodic cognitive testing, confer useful information but may fail to measure subtle changes in readiness that might become meaningful when combined with high workload environments, social strain, or sensorimotor challenges (Sipes and Vander Ark, 2005; Barger et al., 2014; Landon et al., 2015; Slack et al., 2016; Jones et al., 2022). To address these shortcomings, NASA’s Cognition battery represents a significant advancement in cognitive assessment by assessing multiple cognitive domains relevant to astronaut performance (Basner et al., 2015; Moore et al., 2017). The Cognition battery represented an improvement over approaches that solely evaluate single domains because it acknowledged that the operationally relevant cognitive profile of astronauts differs from that of clinical or general-population individuals. However, scheduled periodic testing alone cannot accurately assess higher-order performance demands relevant to exploration-class missions. Hence, monitoring systems implemented in future LDS missions should integrate cognitive examinations with physiological, behavioral, and operational metrics to provide a comprehensive state of mission readiness (Goel et al., 2009; Lim and Dinges, 2010; STD, 2011; Barger et al., 2014; Basner et al., 2015; Landon et al., 2015; Slack et al., 2016; Moore et al., 2017; Piechowski et al., 2024; Abercromby et al., 2025).

In addition, behavioral health monitoring strategies might be operationally difficult to implement because interpersonal dynamics, stress, and mood are difficult to objectively quantify. Furthermore, astronauts may be reluctant to disclose emerging stressors due to cultural factors and/or intrinsic motivation (Sipes and Vander Ark, 2005; Kanas and Manzey, 2008; Kanas, 2015; Slack et al., 2016). Consequently, wearable device technology capable of monitoring physiological stress responses (heart rate variability, electrodermal activity, etc.), sleep patterns, workload, and physical activity may provide valuable adjunctive information (Healey and Picard, 2005; Gjoreski et al., 2016). Wearable devices should be designed and ethically implemented to adhere to mission constraints and be operationally actionable.

Despite its importance to mission readiness, team functioning assessment is similarly underdeveloped. Usually, characteristics of team functioning associated with performance in complex situations such as, cohesion, communication quality, and information flow are assessed retrospectively via surveys, interviews, or debriefings (Cannon-Bowers and Salas, 1993; Helmreich and Merritt, 1998; Salas et al., 2005; Landon et al., 2018; Verhoeven et al., 2022), However, these types of retrospective evaluations are too late for exploration-class missions. For instance, a crew may appear superficially functional while struggling with members speaking up, behavioral support, conflict resolution, or cross-checking that are only visible in certain communication patterns and operational task interactions. Literature from the team sciences and reviews of team risk evaluations suggest that these subtle struggles are where mission-threatening problems may develop (Salas et al., 2005; Landon et al., 2015, 2018; Verhoeven et al., 2022), thereby necessitating a performance-oriented approach. Future systems could assess communication density, response delays, leadership duty assimilation, and changes in social patterns as indicators of behavioral strain rather than waiting for explicit conflict or a catastrophic communication error. In this regard, borrowing concepts from high-performance domains such as aviation, medicine, and military operations, nontechnical skills and team processes are viewed as measurable operational endpoints rather than intangible characteristics (**Figure 4**).

**Figure 4:**
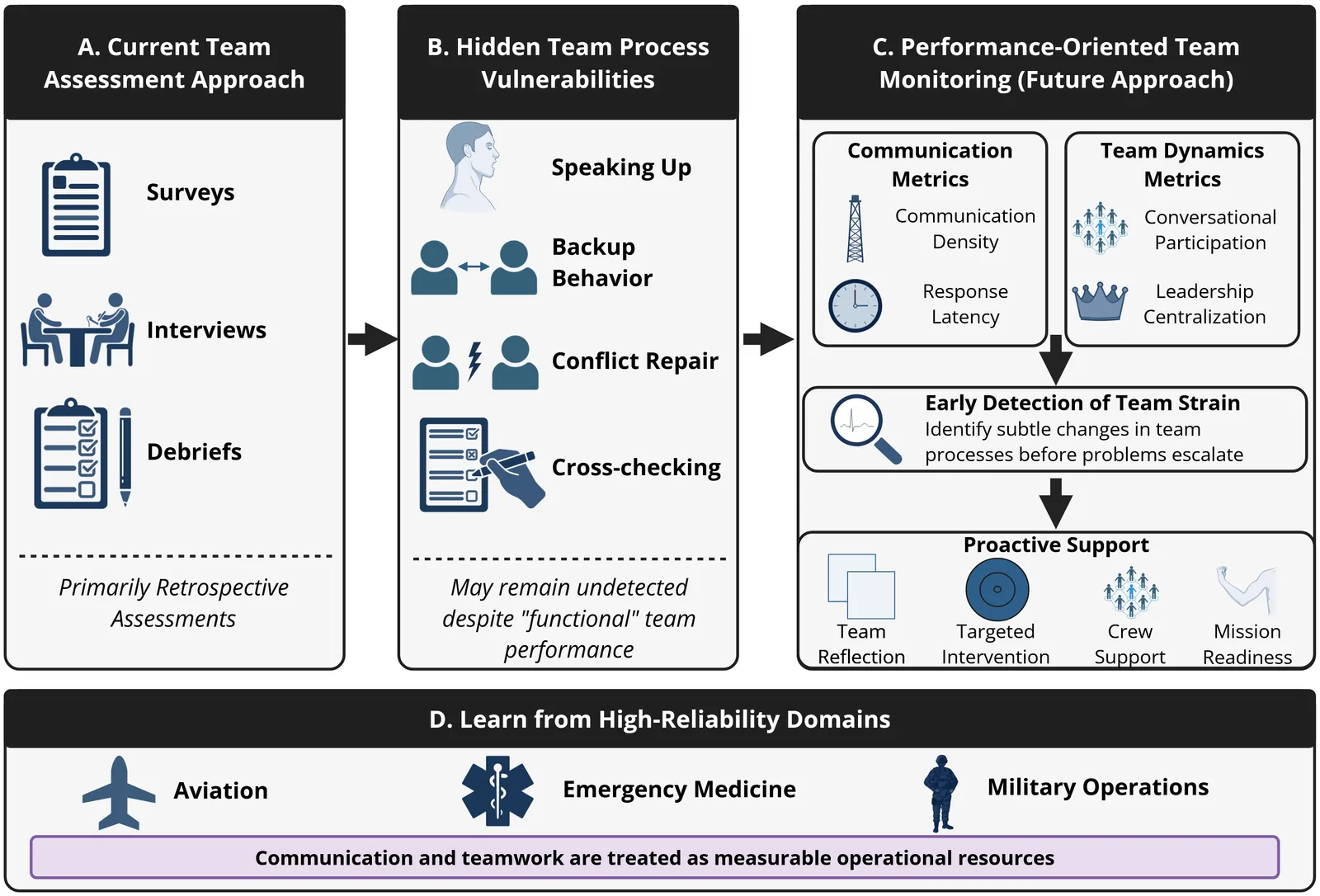
Team dynamics monitoring during spaceflight. Evaluating real-time team dynamics can mitigate declines in interpersonal relationships by adopting a proactive, performance-oriented team monitoring approach that promotes effective communication among crewmembers.

### 4.4 Performance-oriented behavioral readiness strategies

As the human-performance-integrated framework advocates integrating behavioral monitoring strategies, countermeasures must be formulated to reduce pathology while enhancing readiness. Military and sport psychology, along with aviation, provide pragmatic translational models because they focus on maintaining performance under pressure rather than retrospectively addressing stress (Meichenbaum, 1985; Helmreich and Merritt, 1998; Flin et al., 2008; Cornum et al., 2011; Sarkar and Fletcher, 2014; Gross, 2015; Gabbett, 2016; Windt and Gabbett, 2017). In these professions, the most advantageous concepts combine training in realistic scenarios, clear behavioral skills, practical recovery, and continuous feedback about mission readiness status. Just as physical countermeasures are emphasized, psychological skills and preparedness should be trained, rehearsed, monitored, and adapted to mission demands (Helmreich and Merritt, 1998; Gross, 2015; Kanas, 2015; Abercromby et al., 2025).

#### 4.4.1 Stress resilience training

It is important for future crews venturing to the planned lunar base and/or to Mars to recognize that they cannot be trained only for expected, known contingencies that mission control can help resolve. However, they must be trained in operations without external resolution in scenarios in which uncertainty, ambiguity, and delays dominate, along with the psychological stress in which these situations bring. One useful model from high-tempo operational domains is stress inoculation training, which teaches individuals to anticipate stress responses, practice coping mechanisms, and develop skills under controlled conditions (Meichenbaum, 1985). In addition, theories in emotion regulation clarify that adaptive coping in line with stress inoculation is not solely suppressing emotion, but rather the deployment of reappraisal, attentional control, and response modulation strategies relative to the context of the scenario (Gross, 2015). Adopting these approaches can benefit crews of exploration -class or LDS missions, in which maintaining alertness and vigor during atypical operations may depend as much on emotional regulation as on technical proficiency (Meichenbaum, 1985; Arnsten, 2009; Gross, 2015).

Literature from military psychology offers further considerations that suggest resilience is supported by individual coping capabilities along with team-based skills that are embedded within a training culture that promotes adequate feedback systems and shared expectations (Cornum et al., 2011). Therefore, resilience training programs that combine individual mental skills with team reflection, post-scenario reviews, and exposure to realistic mission stressors should benefit LDS crews. Resilience training should be transferable and adaptive, in which crews should be trained in disparate settings that include communication delay, uncertainty, and incomplete information while under fatigue (Meichenbaum, 1985; Cornum et al., 2011; Landon et al., 2015).

#### 4.4.2 Cognitive performance training

In addition to stress-resilience training, cognitive performance training should transition from isolated drills toward conditioning relevant to mission demands, with the primary goal of improving spaceflight-specific cognitive functions (Baddeley, 1992; Endsley, 1995; Wickens, 2008; Miyake and Friedman, 2012; Diamond, 2013). Training to maintain sustained attention, reaction time, and working memory is most likely to be successful when embedded within operational scenarios, especially those that require cognition under fatigue and in moments of stressful demands. In this regard, practitioners can again extrapolate from elite performance frameworks that training with repeatedly provided feedback on limiting performance factors is more likely to produce cognitive adaptation than scenario repetition without context (Sarkar and Fletcher, 2014; Gabbett, 2016; Windt and Gabbett, 2017).

As such, an important target for cognitive performance training is managing cognitive load. In times of extended ICE exposure and uncertainty, future crews will have to identify when workload has exceeded appropriate decision margins and then redistribute duties, accept noncritical activity, and/or simplify procedures without external assistance. This metacognitive skill allows one to realize their shortcomings and requires crew members to monitor their cognitive state and that of each crew member. Through situational awareness entrainment, training programs should teach crews to recognize overload before it becomes apparent after mission or scenario failure (Endsley, 1995; Wickens, 2008; Basner et al., 2015). Wearable devices that enable continuous monitoring of recovery status and/or stress levels might be worth considering to detect overload before it causes mission-level issues.

#### 4.4.3 Team performance training

Team science consistently demonstrates the importance of the aforementioned shared mental models, communication protocols, backup behavior, and adaptive leadership (Cannon-Bowers and Salas, 1993; Salas et al., 2005; Landon et al., 2018). Crews must optimize these capabilities over time during LDS to foster team cohesion and dynamics among crew members. Therefore, team training should include practice sessions with variable leadership roles, conflict resolution, and communication delays or failures so that coordination skills are exercised under realistic social and temporal conditions among the team and not among sole individuals (Landon et al., 2018; Verhoeven et al., 2022).

Concepts from the aviation and human factors literature also outline methods for training nontechnical skills, including briefing interviews, cross-referencing, appropriate challenge of authority, and recovery from communication failures (Helmreich and Merritt, 1998; Flin et al., 2008). For Mars missions, a similar training framework should be adopted and can integrate methods for training in crew resource management, mission autonomy, and self-governance. The framework should also be integrated into pre-flight processes (i.e., astronaut selection and team preparation) and could emphasize role adaptability, conflict repair, and collaborative problem solving in ICE such as LDS (Flin et al., 2008; Landon et al., 2015, 2018; Verhoeven et al., 2022).

### 4.5 Behavioral adaptation across mission phases

Cumulatively, this section outlines the consideration of behavioral readiness training across all mission phases. Pre-flight processes such as astronaut selection, team formation, stress resilience training, and development of inter- and intra-personal communication skills underpin the completion of the subsequent mission in LDS. Considering the integrated performance-supported framework, this phase should also include baseline assessments of and methods to address fatigue and workload awareness and stress management, along with entrainment of how to appropriately self-reflect so that crews can 1) have these skills throughout the mission, and 2) so that individual crew members can identify their own tendencies and operating patterns (Sipes and Vander Ark, 2005; STD, 2011; Kanas, 2015; Landon et al., 2015; Slack et al., 2016; Landon et al., 2018; Abercromby et al., 2025). Emphasis, however, will then shift to maintenance and adaptation during flight. As the mission progresses and reaches terminal processes, sleep management, workload distribution, planned recovery, and access to support resources become increasingly important. Delays in communication during LDS to Mars further augment the need for a transition from ground-directed operations toward crew-based problem solving and identification, collaborative decision-making, and autonomous execution (Sipes and Vander Ark, 2005; Barger et al., 2014; Landon et al., 2015; Slack et al., 2016; Landon et al., 2018; Jones et al., 2022; Verhoeven et al., 2022; Piechowski et al., 2024; Abercromby et al., 2025). As stated above, behavioral training and support should therefore transition from *in-situ* problem detection and adaptive local plan execution (i.e., operational autonomy) to a model centered on executing known procedures for expected contingencies (Landon et al., 2015; Slack et al., 2016; Landon et al., 2018; Abercromby et al., 2025).

Equal attention must be given to post-mission behavioral reconditioning. Astronauts may struggle with reintegration into terrestrial life, experiencing challenges related to circadian entrainment, social role reinstatement, identity transitions, and recovery of cognitive function (Sipes and Vander Ark, 2005; Cornum et al., 2011; Sarkar and Fletcher, 2014; Kanas, 2015; Stahn et al., 2019). Consequently, behavioral rehabilitation is an essential component of long-term astronaut readiness (Sipes and Vander Ark, 2005; Barger et al., 2014; Kanas, 2015).

### 4.6 Implications for exploration missions

Mission success and astronaut readiness are contingent upon behavioral health and cognitive performance. For exploration-class missions, multiple factors discussed above (i.e., circadian disruption, isolation, environmental monotony) influence the executive and social capacities needed for mission success. Future lunar and Mars missions will require integrated behavioral readiness concepts spanning selection, training, evaluation, countermeasures, and post-flight recovery (Cannon-Bowers and Salas, 1993; Helmreich and Merritt, 1998; Salas et al., 2005; Sipes and Vander Ark, 2005; Sandal et al., 2006; Barger et al., 2014; Landon et al., 2015; Pagel and Choukèr, 2016; Slack et al., 2016; Landon et al., 2018; Aeronautics and Administration, 2022; Jones et al., 2022; Verhoeven et al., 2022; Abercromby et al., 2025). Behavioral readiness should be trained and monitored with the same rigor as musculoskeletal, cardiovascular, and sensorimotor readiness to ensure astronauts can sustain decision-making, effective teamwork, and adaptive performance under the constraints of exploration-class missions (**Table 3**).

**Table 3:** Behavioral and cognitive considerations for mission readiness during LDS.

Operational capability	Pre-flight	In-flight	Post-flight	Operational outcome
Psychological Resilience	Implement stress inoculation training, emotional regulation strategies, and graded exposure to realistic mission stressors involving uncertainty and fatigue	Maintain resilience through structured recovery strategies, behavioral support, adaptive coping techniques, and monitoring of stress responses	Reinforce coping strategies, provide psychological support, and facilitate adaptation from mission demands to terrestrial environments	Sustained performance, emotional regulation, and effective decision-making during high-stress operations
Sleep & Circadian Readiness	Establish individualized sleep strategies, fatigue awareness, and circadian preparation before launch	Continuously monitor sleep quality, fatigue, workload, and circadian responses; implement scheduling and recovery strategies to prevent cognitive decline	Restore circadian biology, normalize sleep patterns, and support cognitive recovery	Maintenance of vigilance, reaction time, executive function, and recovery capacity
Cognitive Performance	Train attention, working memory, cognitive flexibility, metacognition, and decision-making using mission-specific simulations	Monitor cognitive readiness and support workload management, task prioritization, anomaly response, and decision-making during high cognitive demand	Assess recovery of cognitive function and progressively restore complex operational responsibilities	Preservation of judgment, adaptability, problem-solving, and mission-critical decision-making
Situational Awareness	Develop recognition of cognitive overload through realistic scenarios requiring multitasking, prioritization, and uncertainty management	Use self-monitoring, team monitoring, and decision-support systems to identify excessive workload and maintain safe cognitive margins	Evaluate cognitive workload tolerance before returning to unrestricted operational activities	Reduced error risk during complex tasks, emergencies, and autonomous operations
Team Performance	Develop shared mental models, communication strategies, conflict management, backup behaviors, and adaptive leadership through prolonged team simulations	Maintain cohesion, communication quality, mutual performance monitoring, distributed leadership, and conflict resolution during isolation and confinement	Conduct structured debriefing, team reflection, and reintegration with operational support systems	Sustained coordination, trust, information sharing, and team effectiveness
Earth-Independent Operations	Train crews using delayed communication, ambiguous failures, resource limitations, and autonomous problem-solving scenarios	Emphasize local issue detection, adaptive planning, collaborative decision-making, and flexible leadership without immediate ground support	Evaluate autonomous decision-making and incorporate lessons learned into future exploration training	Independent mission execution during lunar and Mars operations
Sensorimotor-Cognitive Integration	Prepare crews for altered gravity transitions through integrated sensorimotor, cognitive, and operational task training	Monitor interactions among fatigue, workload, spatial orientation, fine motor control, and task performance during gravity transitions	Restore vestibular function, spatial orientation, manual control, locomotor control, and operational task capability	Safe performance of landing, EVA, navigation, and surface operations
Behavioral & Cognitive Monitoring	Establish individualized baselines for sleep, cognition, psychological state, communication patterns, and team performance	Integrate wearable sensors, cognitive testing, physiological monitoring, communication analytics, and AI-assisted decision support to guide countermeasures	Continue longitudinal monitoring to confirm restoration of behavioral readiness	Early detection of performance decline and individualized intervention
Behavioral Performance Technologies	Utilize VR simulations, cognitive training platforms, and realistic operational rehearsal	Implement VR environments, decision-support tools, passive monitoring, and adaptive countermeasure systems to maintain readiness	Apply technology-assisted recovery and readiness assessment strategies	Continuous optimization of behavioral performance throughout mission phases

### 4.7 SHOP perspective

Behavioral health and cognitive function are fundamental components of operational readiness that must be sustained before, during, and after LDS. Continuous assessment, development, and monitoring of team dynamics, communication, stress resilience, workload and fatigue, sleep metrics, and cognitive function are emphasized to ensure that astronauts can sustain effective mission performance under prolonged exposure to ICE and amid increased autonomy during future exploration-class missions.

## 5 Exercise training and pre-flight conditioning

In addition to training behavioral readiness in the pre-flight phases to enhance mission readiness, exercise training is planned for approximately two years before flight. It is typical for astronauts to experience operational demands that reduce training opportunities in the pre-flight phase. Consequently, efficient training modalities (e.g., microdosing) may be advantageous for maintaining or improving physical fitness when time is limited. Microdosing involves shorter duration, more frequent training sessions with the total weekly volume distributed across an increased number of days and has been reported to be beneficial in military populations and may represent a practical strategy for astronaut training (Kilen et al., 2015; Cuthbert et al., 2024). This practice, however, is separate from ‘exercise snacking,’ which is characterized by short bursts of high-intensity exercise and likely provides insufficient volume needed for astronauts to maintain physical readiness (Rodríguez et al., 2025).

### 5.1 High-performance training regimens

Research that directly assesses the effects of varying training modes, intensities, and volumes in astronauts is somewhat limited. Studies that have been conducted have been generally observational, lacking control groups, and are ground-based analogs (English et al., 2020). Due to this, it is useful to extrapolate from research conducted on athletes and/or military personnel. Alternatively, when evaluating skeletal health outcomes, research in older individuals serves as an excellent source for identifying exercise countermeasures to minimize skeletal impairments during spaceflight or for post-flight rehabilitation.

#### 5.1.1 Aerobic capacity

Recommendations on pre-flight aerobic exercises are currently relatively broad, despite reports suggesting exercise intensity is the primary determinant of cardiorespiratory adaptation (Helgerud et al., 2007; Loehr et al., 2015; MacInnis and Gibala, 2017). Research shows that high-intensity interval training (HIIT), a more time-efficient modality of training, elicits superior improvements in aerobic capacity compared with moderate-intensity continuous exercise (Alansare et al., 2018; Guo et al., 2023). Therefore, HIIT can be implemented as an efficient training method that can enhance pre-flight cardiorespiratory fitness. Additionally, data suggest that astronauts with higher levels of pre-flight aerobic capacity mitigate cardiovascular deconditioning more effectively during flight compared to lesser fit counterparts (Moore et al., 2014). Similarly, the results of the NASA Sprint Study indicate that higher-intensity exercise can preserve fitness when total exercise time is reduced (English et al., 2020). Collectively, these data support implementing high-intensity aerobic training, integrated with lower-intensity exercise, in pre-flight conditioning programs to maximize fitness before flight and to minimize in-flight deconditioning.

#### 5.1.2 Muscle mass & muscle strength

Similar to cardiorespiratory fitness training, it is essential to consider frequency, volume, and intensity when designing resistance training programs during the pre-flight phase. These training principles affect changes in hypertrophy and strength differentially, with resistance load primarily determining changes in strength (Schoenfeld et al., 2015; Refalo et al., 2021; McLeod et al., 2024) and volume determining hypertrophy outcomes (Schoenfeld et al., 2017; McLeod et al., 2024). Interestingly, when training volume is matched, training frequency does not appear to affect strength changes (Cunanan et al., 2018; Kistler-Fischbacher et al., 2021a; 2021b). Conversely, when volume increases simultaneously with training frequency, a dose-response increase in strength is evident (Grgic et al., 2018). For strength development, high loads (≥80% 1-repetition maximum) are required to optimize adaptation; however, in the wake of sufficient and progressive volume, hypertrophic adaptations can occur with low, moderate, or high loads (Schoenfeld et al., 2017; Refalo et al., 2021; Carvalho et al., 2022).

Based on the information above, astronaut muscle mass maintenance is possible if the weekly training volume is sufficiently high. Like aerobic capacity, astronauts who exhibit higher pre-flight muscle mass and strength levels mitigate declines in strength and performance more effectively during flight compared to their counterparts with lower muscle mass and strength (English et al., 2020). However, during-flight logistical issues may create difficulty in maintaining high loads necessary for strength gains. For example, while on Earth, during exercises such as the squat, astronauts lift the external load of the barbell and added resistance along with approximately 85% of their body mass. However, while aboard the ISS, body mass is not lifted during such exercises (Gopalakrishnan et al., 2010), thus resulting in substantially reduced loads to the lower body and contributing to astronaut detraining (Comfort et al., 2021). While the advanced resistive exercise device (ARED) on the ISS provides additional loads that astronauts are familiar with, it is not recommended to load the spine with resistances astronauts are unaccustomed to. Instead, safer alternatives could be increasing loads on single-leg squats or performing a spine-unloaded belt squat, especially for Mars-based missions.

#### 5.1.3 Skeletal health

Preservation of skeletal health is paramount during LDS. Bone loss increases linearly with mission duration and may not fully recover following return to Earth (Gabel et al., 2022) with one study suggesting continued decrements in bone health four years after spaceflight for one astronaut (Matheson et al., 2025). Due to extended mechanical unloading, spaceflight-induced BMD losses occur at more rapid rates compared to those observed during osteopenia and are most pronounced in the lower extremities and spine (LeBlanc et al., 2000; Robling and Turner, 2009; Bailey et al., 2018). Consequently, lower-body and spinal loading should be emphasized in exercise countermeasures to mitigate bone loss.

During spaceflight, the primary osteogenic stimulus is mechanical loading during exercise and can be achieved through a combination of resistance and impact exercises (i.e., plyometrics). Studies involving older individuals with compromised skeletal health show that performing high-intensity resistance training coupled with impact activities effectively enhances BMD (Turner and Robling, 2003; Robling and Turner, 2009; Watson et al., 2015; Harding et al., 2020b, 2020a; Kistler-Fischbacher et al., 2021a; 2021b) Moreover, microdosing resistance exercise could influence skeletal adaptation through distributing osteogenic stimuli across frequent training sessions during flight. Collectively, these findings suggest that training programs designed to enhance strength and power development also support bone integrity before and during spaceflight.

### 5.2 Periodization & programming and load management

Periodization and planning are often confused with each other (Cunanan et al., 2018). Programming is the selection of training frequency, exercises, sets, repetitions, and intensity over blocks of training, while periodization is similar planning but over a much longer time period and considers the additional stressors to which the individual may be subjected, in addition to the competition schedule in athletes (Cunanan et al., 2018). Planning astronaut training does not have to consider tapering and peaking at specific points for competition per se, yet there will be specific periods of time in mission preparation where time becomes limited, or the physical or psychological stress of specific aspects of mission preparation increases. In athletes, unplanned training volume or competition frequency, along with periods of heightened psychological stress, increases the risk of musculoskeletal injuries (Moore et al., 2014; Mølmen and Rønnestad, 2024; Tranaeus et al., 2024). Theoretically, a similar phenomenon can occur in astronauts subjected to high workloads (i.e., repeated EVAs). However, quantifying workloads in research has been inconsistent and called into question, which may affect astronaut-related implementations (Udby et al., 2020; Kalkhoven et al., 2021). As such, in the pre-flight phase, periods of increased physical and/or psychological stress should be accounted for in the long-term planning of astronaut training, with weekly training volume being reduced accordingly, by simply reducing the total number of sets performed in a training session. Although, importantly, this should not be at the expense of appropriate exercise intensity to ensure adaptation or maintenance of a specific physical quality.

In-flight training could also integrate periodization concepts by accounting for periods of increased psychological or physical demands (e.g., planned EVAs). Additionally, as future mission priorities might reduce time for daily exercise, one solution is to shorten exercise bout duration but increase intensity, especially since intensity primarily drives adaptation and mitigates declines. Post-flight training can also be similarly altered (discussed below), depending on the astronaut’s magnitude of multisystem deterioration upon return to Earth and while accommodating the changes in spinal characteristics (Bailey et al., 2018; Burkhart et al., 2020; Bailey et al., 2022) due to adjustment to Earth’s gravity.

#### 5.2.1 Aerobic capacity

Building on the cardioprotective benefits of pre-flight aerobic fitness noted above, programming could balance continuous exercise with HIIT, which has been shown to enhance aerobic capacity, even in well-trained individuals (Helgerud et al., 2007; Seiler et al., 2013). An additional benefit of HIIT is that it can offset the mixed signaling effect of concurrent training, thereby mitigating any reductions in hypertrophy or strength adaptations from resistance exercise that could potentially be blunted in the presence of steady-state aerobic training (Wilson et al., 2012; Methenitis, 2018).

Prolonged steady-state exercise could be implemented in periods where exercise time is not restricted. However, programming HIIT during periods when time is limited would be a more efficient and potentially effective strategy. HIIT will also complement heavy strength training, which results in shorter-duration, high-intensity, resistance training sessions.

#### 5.2.2 Muscle mass & muscle strength

Ideally, muscle mass and muscle strength should be emphasized in separate training blocks, as focused adaptive stimuli differ for each. Moreover, one phase can exert benefits on the other. For example, the increased muscle mass associated with hypertrophy training facilitates a propensity for increases in force production during the strength phase. Conversely, increased strength gained during a block of strength training results in higher loads being able to be incorporated into the hypertrophy phase, which facilitates increased training volume (DeWeese et al., 2015a, 2015b; Haff, 2024) (**Table 4**). Another advantage of high-load strength training is the beneficial effects of increased BMD that come as a result (Kistler-Fischbacher et al., 2021a; 2021b).

**Table 4:** Exercise considerations to optimize mission readiness and rehabilitation.

Operational capability	Pre-flight	In-flight	Post-flight	Operational outcome
Aerobic Capacity	Maximize VO_2_peak prior to launch w/steady state or HIIT. Prioritize higher-intensity approaches when time constraints exist to improve training efficiency	Maintain using individualized HIIT, threshold training, cycling, rowing, etc. Emphasize sufficient intensity to prevent cardiovascular detraining	Gradually restore upright exercise tolerance w/progressive aerobic reconditioning (Zone 2 → threshold → HIIT) based on orthostatic tolerance	Preservation of aerobic capacity, EVA capability, workload tolerance, and rapid recovery during operational tasks
Muscular Strength	Develop maximal strength via high-load RT (≥80% 1RM); multi-joint exercises (squat, deadlift, press, pull variations) and lower-body/trunk strength are emphasized	Maintain mechanical loading w/HIRT, eccentric-focused training, unilateral movements, and individualized load adjustments based on strength monitoring	Progressively reload tissues w/phased RT: movement restoration → HT/strength rebuilding → RFD	Maintenance of force production, equipment handling capability, mobility, and ↓ injury risk
Skeletal Muscle Hypertrophy	↑ muscle mass through HT blocks, emphasizing volume (e.g., moderate loads, higher repetitions) to maximize before launch	Preserve muscle size through adequate training volume, progressive overload strategies, and monitoring of muscle morphology (e.g., ultrasound assessment)	Restore lost lean mass through progressive HT-focused training, nutrition support, and individualized loading strategies	Preservation of functional capacity and mitigation of muscle atrophy
Skeletal Health & Bone Loading	Maximize BMD via HLRT, impact activities, and progressive osteogenic loading strategies	Maintain bone structure using frequent mechanical loading, RT, and available impact/loading countermeasures	Gradually restore axial loading tolerance before progressing to HLRT and high-impact activities	Maintenance of bone integrity, fracture resistance, and long-term skeletal health
Power	Incorporate explosive training (jumps, throws, Olympic lifting derivatives) after adequate strength development	Preserve NM function using VBT or explosive movements equipment permitting	Progress from basic movement quality to power training, acceleration/deceleration, agility	↑ reaction time, proprioception, EVA performance, and emergency response
Periodization & Load Management	Implement structured training phases emphasizing HT, strength, power, and aerobic development while adjusting workload during mission-preparation periods	Use flexible programming and exercise microdosing when operational demands limit available training time while preserving intensity	Progress rehabilitation based on functional milestones, adjusting loading based on recovery status	Optimized adaptation while ↓ overtraining, injury risk, and performance decline
Sensorimotor & Neuromuscular	Development of balance, coordination, trunk stability, proprioception, and functional movement competency	Integrate NM control challenges to mitigate proprioception deconditioning	Restore gaze stability, vestibular function, postural control, proprioception, agility, and reaction time through phased rehabilitation	Improved post-landing recovery, locomotion, EVA readiness, and operational movement quality
Virtual, Augmented Reality Training	Use VR/AR environments for task-specific preparation, cognitive-motor integration, and mission simulation	Combine VR with exercise systems to improve motivation, adherence, psychological state, and environmental stimulation during confined missions	Apply VR-based rehabilitation to restore balance, gait, coordination, and operational task performance	Enhanced training engagement, sensorimotor adaptation, and psychological resilience
Wearables and Performance Monitoring	Establish individualized baselines for CV fitness, strength, movement quality, sleep, and readiness	Continuously monitor physiological responses using wearable sensors (HR, HRV, EMG, IMUs, biomarkers) to guide individualized countermeasures	Use objective monitoring to guide progression, identify persistent deficits, and confirm return-to-readiness	Real-time individualized countermeasure adjustment and performance optimization
Mission-Specific Conditioning	Tailor preparation according to mission duration and demands; longer missions require greater pre-flight optimizations in aerobic capacity, strength, muscle mass, and BMD	Adjust countermeasures according to μG exposure, habitat limitations, partial G environments, and available equipment	Individualize recovery based on mission duration, physiological decline, and operational requirements	Sustained capability across increasingly demanding exploration missions

During LDS, it would be beneficial to monitor muscle thickness or cross-sectional area (i.e., ultrasound) and muscle strength (i.e., mounted strain gauge) to assess the catabolic effects of μG and the effectiveness of countermeasures. Due to individual responses to spaceflight, this would be advantageous for determining individualized exercise regimens to account for any changes from the previous training phase.

#### 5.2.3 Skeletal health

Given the limited BMD recovery discussed above, it is of great importance to minimize reductions in BMD during flight. Inherently, maximizing BMD in pre-flight training would be a prudent approach. Doing so also complements approaches to maximizing muscular strength (Kistler-Fischbacher et al., 2021a; 2021b) and optimizing aerobic capacity, since impact activities can improve BMD (Kistler-Fischbacher et al., 2021a; 2021b).

One principle to strongly consider in this regard is progressive overload, which is essential for training adaptations. However, monitoring the combined load when all activities are progressed simultaneously is recommended. Therefore, one aspect of training can be progressively overloaded while the other is maintained. For example, emphasizing strength development for 4–6 weeks while maintaining aerobic capacity, followed by 4–6 weeks of strength maintenance, while emphasizing increased aerobic capacity. Alternating such areas of emphasis would still enable osteogenic stimulation initially from increased axial loads during strength training. In contrast, impact loads could increase via higher running speeds or longer durations.

### 5.3 Differences in training in preparation for LEO, moon, and mars missions

Planned mission duration is a central factor to consider when preparing for LDS due to the relationship between bone degradation and mission length (Gabel et al., 2022), which would likely be similar for aerobic capacity and muscle mass as well. Therefore, it is important to maximize BMD, aerobic capacity, muscle mass, and strength to a greater degree for longer-duration missions compared to shorter ones.

Astronauts in LEO will continually experience μG, while astronauts who are moon or Mars bound will have to adapt to an altered gravitational vector. However, the demands of mission tasks in these environments are currently unknown and can only be theorized. Although, compared to Earth-bound individuals, reduced gravity will result in substantial decreases in musculoskeletal loading, which would decrease musculoskeletal health and function as have been observed in LEO, albeit to a lesser extent if mission duration were comparable. As such, effective exercise countermeasures will remain essential on the moon or Mars. Adequate countermeasures strategies in these environments have yet to be determined but will be identified in the coming years. In addition, astronauts will experience μG while transporting to and from the moon and Mars, which necessitates countermeasures similar to those used in LEO, which still need improvements.

### 5.4 SHOP perspective

Accordingly, the SHOP framework recommends that pre-flight exercise programming prioritize maximizing physical fitness before launch through individualized, periodized training that develops aerobic capacity, muscle hypertrophy, maximal strength, and osteogenic loading. HIIT should be integrated with heavy resistance and impact-loading exercise to optimize cardiovascular fitness, musculoskeletal performance, and bone health, while flexible programming strategies (e.g., microdosing training) should be implemented during periods of increased operational demands to preserve training intensity and maintain mission readiness.

## 6 In-flight performance support

### 6.1 Application of flexible wearable technology

Missions to the moon and Mars will require continuous monitoring of astronaut performance and health to promote optimal health and mission success (Buckey et al., 1996). Therefore, researchers have been examining methods to enable continuous monitoring astronaut health and performance in the form of wearable devices (Wang et al., 2025).

Wearable and flexible sensing systems are lightweight, noninvasive, capable of continuously monitoring physiological responses with limited crew burden, and can adhere to overall mission constraints. These are important characteristics to consider for exploration-class missions. In addition to supporting health during flight, wearables may also support individualized recovery and rehabilitation following mission completion. A wide range of physiological variables can be monitored with currently available wearable technology. Cardiovascular function and hemodynamics can be monitored by photoplethysmography- and electrocardiography-capable wearable devices (Mundt et al., 2005; Charlton et al., 2023). For musculoskeletal and sensorimotor adaptations, devices capable of utilizing electromyography and inertial measurement unit technology can be used. Wearable eye-tracking technologies, along with electroencephalography, allow assessment of vestibular and cognitive functions. Also, for clinical and/or nutritional status assessments, analyzers that measure biological samples (i.e., blood, sweat) can monitor stress, inflammation, and hydration (Gao et al., 2020; Zhang et al., 2020).

By implementing the use of wearables, astronauts can obtain early detection of performance or health decrements, cultivate individualized countermeasures, and enhance decision-making. By combining the various metrics that wearable technology provides, astronauts and specialists alike can receive a more comprehensive outlook of astronaut readiness (Bellisle et al., 2020; Wang et al., 2025; Khan et al., 2026).

However, before wearable technology can be utilized in LDS, challenges remain in its implementation. Devices must be resilient to radiation, be sustainable in extreme conditions, and survive prolonged exposure to μG (Wang et al., 2025; Khan et al., 2026). Cybersecurity, data handling and privacy, and interoperability also must be addressed to ensure that wearable-derived data can be utilized to make informed decisions that may influence mission outcomes. Additionally, health and performance data analytics will have to be operated locally, onboard, due to significant communication delays and autonomous operations. Computationally, this provides significant obstacles to health monitoring. However, these issues are currently being addressed via NASA’s implementation of a high-performance spaceflight computing processor, a computing system that enables significantly enhanced computing power compared to historically used systems (Powell, 2018). Cumulatively, once these issues are addressed, a unified platform that integrates multimodal health and psychological metrics can be integrated (Wang et al., 2025).

As wearable technologies continue to evolve, they are expected to transition from passive monitoring devices to integrated health-management systems capable of detecting risk, informing countermeasure implementation, and supporting Earth-independent medical operations (Wang et al., 2025). Consequently, wearable technologies will likely serve as foundational components of future astronaut readiness regimens designed to preserve health, optimize performance, and sustain human presence in LDS to Mars.

### 6.2 Individualized exercise countermeasures

Countermeasures for interplanetary travel are critical for minimizing musculoskeletal atrophy, particularly given the increasing durations of proposed spaceflight missions. However, the individualization of such countermeasures is likely difficult to identify, especially given that much of the research to date suggests that existing exercise countermeasures for LDS are insufficient to eliminate the deleterious effects of μG on the musculoskeletal system (Comfort et al., 2021). In fact, historically, the International Space Station (ISS) exercise countermeasures were found not to completely protect astronauts from the deleterious effects of μG on the musculoskeletal and cardiopulmonary systems (English et al., 2020). A systematic review and meta-analysis (Comfort et al., 2021) found large decreases in lower-limb muscle force production following LDS, irrespective of the exercise countermeasure, suggesting that the exercise countermeasures currently used by astronauts are insufficient to maintain muscle function and mass. Additionally, cumulative axial loading is altered in μG, which presents challenges in ensuring proper lower-body exercise mechanics during LDS. To combat this, especially for longer duration missions, NASA’s Human Research Program Advanced Exercise Concepts initiative functions to evaluate more compact exercise alternatives (e.g., flywheel-based devices, pneumatic resistance systems, closed-loop devices) (Perusek et al., 2015). Flywheel/inertial systems are particularly relevant, as they generate resistance through eccentric braking against angular momentum rather than stacked mass, and have produced musculoskeletal adaptations comparable to ARED and free-weight training in simulated μG studies (Berg and Tesch, 1998; Owerkowicz et al., 2016; Chaabene et al., 2022). Additionally, authors recently noted that current exercise recommendations call for combining strength and aerobic training (i.e., concurrent training); however, these training strategies may not be implemented properly (Jones et al., 2019; Comfort et al., 2021). When developing exercise protocols for μG, it is essential to consider time limitations and practicality. During spaceflight, strength training should be conducted prior to aerobic training to minimize effects sometimes shown by concurrent training. Emphasizing eccentric-focused strength training enhances mechanical load with a reduced metabolic constraint (Dudley et al., 1991; Jones et al., 2019) (**Table 4**). To preserve aerobic capacity, options like maintenance cycles or HIIT on a rowing machine are recommended. Moreover, cycle ergometry and rowing may be preferable to treadmill running. However, the need for more personalized exercise countermeasures remains, especially to improve exercise prescriptions for crew members during extended spaceflight missions.

### 6.3 Virtual reality training during LDS

Virtual reality (VR) training has become a method for simulating environments to support training in specific settings. Advancing the frontier of human spaceflight inherently requires novel and creative solutions, such as VR training, to mitigate the deleterious effects of μG on health, performance, and overall vitality. ISS data indicate that astronauts typically exercise for approximately 2 hours per day, approximately 6 days per week, using various modalities such as cycle ergometers, treadmills, and resistance exercise devices. However, trans-lunar and planetary missions will not permit generous volume and mass allotments from exercise systems to cover these exercise modalities, necessitating novel systems that can be leveraged. Some have proposed VR training as a potential solution, mainly from the perspective that if only one exercise modality can be performed due to space limitations, a VR system that can help immerse the individual in a wide variety of settings could be a helpful way to encourage more consistent use and potentially improve one’s perception of well-being (Keller et al., 2022b). One recent trial showed that integrating a VR scenario into the SPRINT protocol, using a flywheel ergometer, resulted in greater improvements in motivation, affect, and mood restoration than exercising without VR in healthy males (Keller et al., 2022b). It is generally believed that combining exercise protocols with VR could enhance both physical fitness and psychological experience. However, studies in true spaceflight are needed to address this as neurovestibular responses are altered in μG and combining an intensely visuospatial stimulation via VR could, theoretically, exacerbate space adaptation syndrome.

### 6.4 SHOP perspective

The SHOP framework recommends that in-flight exercise countermeasures evolve from standardized exercise prescriptions to precision human performance programs that continuously adapt to each astronaut’s physiological status and operational demands. Training should prioritize preservation of maximal strength, lower-body and spinal loading, aerobic capacity, and musculoskeletal integrity through individualized concurrent training, with resistance exercise performed before aerobic exercise, strategic incorporation of eccentric and HIIT, and integration of emerging technologies, such as VR and physiological monitoring, to optimize adherence, training quality, and mission readiness.

## 7 Post-flight physiological and psychological adaptation to spaceflight

Return to Earth following spaceflight requires astronauts to rapidly re-adapt from μG or partial-gravity environments to Earth’s gravitational load. This gravitational transition (g-transition) imposes substantial physiological and psychological demands that may compromise functional performance immediately after landing. Consequently, post-flight recovery should not be viewed solely as a reversal of deconditioning, but rather as a structured process aimed at restoring functionality and operational readiness for subsequent activities of daily living and future missions.

### 7.1 Cardiovascular and neurovestibular recovery

Restoring cardiovascular functionality is one of the most immediate challenges post-flight. LDS results in plasma volume reductions, cardiac mass atrophy, autonomic dysfunction, leading to orthostatic intolerance (Diedrich et al., 2007; Deepika et al., 2021; Jordan et al., 2022; Khune, 2024). With these adaptations, astronauts are likely to experience pre-syncope or syncope symptoms for a time after landing. It is common for astronauts to experience orthostatic intolerance following LDS and shorter missions. Although recently, post-flight cardiovascular responses have improved with adjunctive countermeasures (i.e., gradient compression garments) (Lee et al., 2025).

Naturally, reduced cardiovascular function predicates decreased cardiorespiratory fitness (Vernice et al., 2020; Scott et al., 2021; Rivas et al., 2023). Recovery after LDS may require one month or longer for aerobic capacity to be restored, with astronauts exhibiting substantial inter-individual variability compared to short-duration mission recovery (Sponbeck et al., 2021; Varanoske et al., 2023). Thus, highlighting the importance of maximizing pre-flight aerobic capacity to mitigate performance during flight, thereby protecting cardiovascular function post-flight.

In addition to cardiovascular considerations, sensorimotor and neurovestibular impairments offer post-flight challenges. Astronauts typically experience disorientation, manual control deficits, and impaired balance following landing due to central nervous system adjustments (Wood et al., 2022). These adaptations may impair functional tasks conducted post-flight such as ambulation, vehicle egress, and manual task performance. Microgravity exposure induces a cephalic fluid shift and is associated with post-flight increases in gray matter volume in the superior brain and a reduction in gray matter volume at the base of the brain. Although most symptoms resolve within one to two weeks, persistent changes in brain structure, cerebrospinal fluid dynamics, and sensorimotor integration have been documented following long-duration missions. Changes in cerebrospinal fluid dynamics can result in ventricular expansion of up to 25% compared to pre-flight volumes. The mechanistic links between changes in brain structure and sensorimotor or neurovestibular function are an active area of investigation (Roy-O’Reilly et al., 2021). Spaceflight-associated neuro-ocular syndrome (SANS), characterized by structural ocular changes and visual disturbances, may also persist for months to years after landing, highlighting the need for continued longitudinal monitoring (Macias et al., 2020; Buckey and Lan, 2025).

#### 7.1.1 Virtual reality and artificial reality training post-flight

VR and artificial reality have been demonstrated to be successful during neuromuscular rehabilitation in stroke patients and individuals with cognitive impairments, improving gait, dynamic balance, and postural stability (Lorkowski et al., 2025). As gait, balance, and postural stability have all been shown to be compromised during the post-spaceflight period in astronauts, there may be benefits to using virtual, augmented, or artificial reality training before return to Earth, although this requires further exploration. Interestingly, Lonner and colleagues (Lonner et al., 2023) also demonstrated that VR may be an effective countermeasure for reducing post-flight motion sickness.

Lack of motivation and reduced adherence to exercise recommendations diminish the effectiveness of any exercise prescription in any setting. Therefore, Keller and workers (Keller et al., 2022a) recently demonstrated that using VR to augment flywheel training for two weeks enhanced motivation and mood, although there were no differences in physiological markers. These results show that alternative modalities of exercise, such as VR, can enhance motivation and compliance with exercise recommendations. However, longer-duration interventions are needed to assess long-term reliability and to determine if effects on exercise or functional performance occur.

### 7.2 Musculoskeletal recovery

Musculoskeletal atrophy and degraded functionality remain hallmark characteristics of prolonged exposure to μG. Following LDS, astronauts commonly experienced decreased lean body mass and muscle strength, despite advances in exercise countermeasures over the past 30 years (Greene et al., 2021). Restoring levels of lean body mass to those of pre-flight levels can take multiple years, thereby highlighting the importance of mitigating such decreases during flight. However, the magnitude of these changes exhibits considerable inter-individual variability, with lower-extremity and trunk musculature generally experiencing greater losses than upper-extremity musculature. Faster rates of recovery have been reported with intensive post-flight reconditioning programs and strong crew compliance with lifestyle-related interventions, including personalized nutrition and exercise prescriptions (Petersen et al., 2017). Reductions in muscle strength of approximately 10–30% have also been reported following spaceflight, particularly in the knee extensors and trunk musculature (Williams et al., 2009; English et al., 2015; Comfort et al., 2021; Fiedler et al., 2024; Lee Satcher et al., 2024). Although intensive post-flight rehabilitation often restores strength toward baseline values within several weeks, residual deficits are common (English et al., 2015).

Morphological adaptations also occur within the musculotendinous unit (Roberts et al.; Roffino et al., 2021). Reductions in muscle cross-sectional area, tendon stiffness, and connective tissue integrity have been documented following prolonged unloading and may increase injury risk during gravitational reloading (Scott et al., 2021). These pathological changes not only reduce the tensile strength of the tendon but may also increase the risk of musculotendinous injuries during readaptation to Earth’s gravity. One approach to assess these morphological changes is ultrasound-based assessments, because structural changes often precede noticeable declines in functionality (Seynnes et al., 2007; Sponbeck et al., 2021).

Weight-bearing tissues (i.e., bone, cartilage) are particularly susceptible to the effects of μG. BMD losses of approximately 1–1.5% per month have been reported during long-duration missions despite contemporary exercise countermeasures (Sibonga et al., 2024). Though loss of skeletal muscle mass in-flight has improved with modern exercise prescriptions and hardware, these changes, including the implementation of ARED, do not appear to preserve BMD better (Comfort et al., 2021). Moreover, a strong relationship exists between mission duration and skeletal loss, with trabecular bone appearing especially vulnerable (Sibonga et al., 2024).

Although some aspects of skeletal recovery occur following return to Earth, persistent deficits in bone microarchitecture have been observed years after LDS (Sibonga et al., 2024). Bone turnover biomarkers remain increased in plasma for several months post-flight, with recovery rates differing between cortical and trabecular bone (Niehoff et al., 2016). In addition, cartilage metabolism may also be influenced by spaceflight, though the long-term significance of potential changes has yet to be delineated (Niehoff et al., 2016). Collectively, these findings suggest that continued research is needed for strategies that are capable of preserving skeletal health during exploration-class missions.

### 7.3 Operational reconditioning following spaceflight

Integrating concepts from aerospace medicine, human performance, behavioral health, and strength and conditioning expertise is essential for optimizing reconditioning following LDS. Within the proposed framework, rehabilitation following landing must be individualized, impairment-driven and centered on restoring operational and functional capacity. Key principles of this plan include: (1) comprehensive evaluation of physiological and behavioral decrements; (2) inclusion of interdisciplinary collaborators; (3) implementation of phased, goal-oriented, and adaptive rehabilitation strategies; and (4) continuous assessment of functional outcomes that enable guiding progression. Progression through the rehabilitation period should be based on movement quality restoration, return of physical capacity and cognitive readiness, and completion of pertinent functional tasks. Accordingly, recovery should be viewed as an extension of the astronaut operational readiness continuum rather than an isolated endeavor. Ultimately, the objective is not solely recovery from LDS but restoring capacities essential for future mission performance.

### 7.4 Impairment-driven assessments with functional orientation

Postflight assessments are targeted to identify specific movement system deficits and are structured to begin immediately upon return to Earth’s gravity. Astronauts are evaluated by the mission-assigned crew surgeon, who performs a comprehensive physical assessment and will render field care as necessary (e.g., treatment for motion sickness, orthostatic intolerance). NASA’s Spaceflight Standard Measures and pre- and post-flight medical assessments (ex. Medical B exams) are also completed, and this program provides a structured, cross-disciplinary framework for understanding human adaptation to μG (Cranford, 2023). These measures are designed to deliver a minimal yet meaningful dataset that characterizes physiological, psychological, and biological responses to μG and associated mission stressors. The program integrates various domains into a monitoring strategy that includes sleep, cognitive and neurobehavioral function, physiological and biochemical status, microbiome sampling, cardiovascular structure and function, musculoskeletal health, and sensorimotor and postural control evaluations (NASA, 2017; Lee et al., 2019; (OCHMO), 2023).

Various diagnostic procedures are used to assess cardiovascular structure and function, such as imaging techniques (i.e., carotid intima-media thickness ultrasound), orthostatic assessments to measure baroreflex sensitivity and cardiovascular remodeling, and aerobic capacity via a peak aerobic capacity test (Lee et al., 2019). Evaluations to measure sensorimotor, proprioceptive, and postural control include gait assessments, balance tests, computerized dynamic posturography, and vestibular-ocular examinations. Musculoskeletal health is evaluated through various exams. These include isokinetic strength testing, functional fitness tests, such as isometric mid-thigh pull, isometric bench press, agility tests, a weekly mobility, activation, and postural control assessment, spinal magnetic resonance imaging (MRI), and a whole-body dual-energy X-ray absorptiometry scan (DEXA) (Cranford, 2023). Though each of these assessments is performed at certain intervals at all mission phases, most are completed within the first week following landing and subsequently repeated weekly, then 30 days and 360-days post-flight.

### 7.5 Interdisciplinary integration and phase-based reconditioning

Optimal recovery following LDS requires an integrated, interdisciplinary approach that aligns medical safety with restoration of operational performance (Association, 2024). Similar to contemporary models used in elite sport and rehabilitation medicine, post-flight reconditioning should involve coordinated collaboration among operational flight surgeons, physical medicine providers (e.g., physical therapists and athletic trainers), strength and conditioning specialists, behavioral health professionals, and other human performance disciplines. This approach recognizes that successful recovery extends beyond reversal of physiological impairments and ultimately aims to restore operational readiness.

NASA’s ASCR and Musculoskeletal Medicine and Rehabilitation (MSK) programs provide an example of this interdisciplinary approach through a phased reconditioning model consisting of Reset, Rebuild, and Refine phases (**Table 4**) (Cranford, 2023). Although progression typically occurs over approximately 45 days, advancement is individualized based on continuous assessment of physiological recovery, functional performance, and mission requirements.

#### 7.5.1 Reset phase

The Reset phase emphasizes restoration of physiological stability and movement quality. Restoring postural stability and proprioception, alongside recovery of gaze stability and orthostatic tolerance, is the primary goal of this phase. To achieve these goals, astronauts are directed to perform upright or semi-recumbent low-to-moderate intensity aerobic exercise (for aerobic capacity), balance training, and neuromuscular facilitation exercises (Horak, 2009; Bloomberg et al., 2015; Hides et al., 2016; Hain et al., 2018; Laursen and Buchheit, 2019; Lee et al., 2019; Macaulay et al., 2021). Progression during this phase focuses on improving sensorimotor control and reestablishing tolerance to Earth-bound gravity.

#### 7.5.2 Rebuild phase

The Rebuild phase gradually reintroduces mechanical and gravitational loading while continuing to reinforce sensorimotor adaptations established during the Reset phase. Using National Strength and Conditioning Association (NSCA) principles, strength training initially prioritizes movement quality, neuromuscular control, and tissue tolerance using stable, multi-joint movement patterns before progressing to greater loading complexity and intensity (Haff and Triplett, 2015; Haff et al., 2016). Aerobic reconditioning continues to prioritize low-impact modalities, progressing to higher-intensity intervals (HIIT) and lactate threshold (LT) training based on the astronauts’ returning VO_2_ max assessments (Lee et al., 2019). Collectively, these interventions establish the physiological foundation necessary for restoration of preflight fitness and functional capacity.

#### 7.5.3 Refine phase

In this final phase, refining functional capacity and readiness is the primary focus. Training prioritizes skill-related fitness components such as power, balance, and agility in addition to completing complex tasks. Power training is essential for improving reactive balance and overall load tolerance. The NSCA recommends 70–80% of 1RM for multi-joint lifts with 3–6 sets of 3–6 repetitions (Comfort et al., 2023). Rest periods of 2–3 minutes allow full recovery, maximizing velocity and neural output (Cormie et al., 2011). As sensorimotor function improves, rehabilitation should progress to include open- and closed-loop agility and reactive balance training to restore dynamic stability and prepare astronauts for the complex movement demands associated with operational tasks (Nimphius et al., 2018). Functional activities may include load carriage, directional change, uneven terrain ambulation, and mission-specific task simulations designed to prepare astronauts for occupational demands. These strategies are incorporated to accelerate the return to full spaceflight physical readiness (Lee et al., 2019). The ultimate objective of this phase is restoration of mission-relevant performance capabilities rather than simply normalization of isolated physiological variables.

### 7.6 Goal-oriented and adaptive reconditioning

Goals are created jointly with the astronaut and full interdisciplinary team and are specific, measurable, attainable, relevant, and time-bound (SMART). Before completion of the in-flight mission phase, astronauts work with the human performance team to develop short- and long-term goals targeting both the immediate postflight phase (30 days) and the full one-year post-flight. These individual goals are taken into consideration and progress from physiological recovery to personal goals, and ultimately to full operational readiness (e.g., a fully mission-qualified astronaut)

### 7.7 Continuous outcome monitoring and progression

Standardized metrics (e.g., vestibular function, functional fitness assessment, etc.) are routinely measured and used across the team to track daily, weekly, and monthly progression. Additionally, astronauts are assessed daily on perceptions of functionality, perceived exertion, and fatigue level. These metrics are used to continually refine targeted interventions tailored to the astronaut’s needs. This ongoing monitoring allows for tailored progression of needs, which may vary across sensorimotor, strength, and cardiovascular domains. Additionally, this type of monitoring will identify vulnerabilities that require continued interventions. Lastly, these metrics serve to ensure a return to prior levels of fitness and function, enabling a return to full mission readiness.

### 7.8 Short- versus long-duration mission considerations

Mission duration primarily influences the rigor of post-flight rehabilitation. Short missions confer less severe physiological adaptations, enabling faster progression and quicker return to operational duties (LeBlanc et al., 2000; Watson et al., 2015; Lee et al., 2019; Watson et al., 2019; Harding et al., 2020a, 2020; Kistler-Fischbacher et al., 2021b; 2021a). Contrarily, LDS is associated with larger reductions in musculoskeletal health and sensorimotor and cardiovascular impairments, thereby necessitating slower progression through the program and more involved oversight from medical personnel (Macaulay et al., 2021). Therefore, rehabilitative/reconditioning planning should be individualized and centered around mission durations, health and fitness status, and operational requirements.

### 7.9 Parallels with return-to-play models in elite athletes

Restoring readiness in astronauts post-flight is like restoring return-to-play in elite athletes. Successful return-to-activity requires structured rehabilitation phases that focus on restoring physical capacity, movement quality, psychological readiness, and, inevitably, performance. As used in sports medicine rehabilitation models, advancement through recovery should be based on objective functional criteria rather than time alone.

Currently, decisions on return-to-flight status are dependent on health assessments, medical standards, and clinical judgment. However, future missions necessitate the development of operational-focused performance standards that inform readiness for mission-critical tasks (i.e., EVAs). Establishing these thresholds, particularly under partial-gravity conditions, represents an important area for future research and will be essential for supporting safe and effective human exploration beyond low-Earth orbit (Ryder et al., 2019; Moudy et al., 2025).

### 7.10 SHOP perspective

The SHOP framework recommends that post-flight reconditioning be managed using a phased, interdisciplinary, and impairment-based model that restores operational capacity. Rehabilitation should restore multisystem health and function, then progress to develop power, agility, balance, and functional task execution. Alternative methods of training, such as VR, AI-supported systems, and augmented reality, can also be incorporated. Advancement should be individualized using continuous physiological and functional monitoring, with return-to-flight or unrestricted operational activities determined by achievement of objective performance milestones rather than time elapsed since landing.

## 8 Conclusion

The integration of medical-driven human performance and aerospace medicine is not merely advantageous; it is a non-negotiable element for mission success, especially as we look toward Mars and beyond. Space missions, particularly those lasting years, will challenge the human body and mind in unprecedented ways. These extreme conditions, spanning continued microgravity occupancy in LEO, partial gravity on the Moon, and the operational and environmental stressors anticipated on Mars, demand an integrated approach that protects health while sustaining mission-relevant human-system capability.

This paper advances a unifying framework for Spaceflight Human Optimization and Performance by positioning astronauts within an astronaut–athlete model and linking that paradigm to practical systems for monitoring, countermeasure delivery, and mission-phase decision-making (**Figure 5**). Across physiological conditioning, nutrition, behavioral health, and performance support, the goal is not simply to “maintain fitness,” but to preserve—and when needed, restore—readiness through structured preparation, focused countermeasures, and coordinated post-mission reconditioning. In doing so, the framework emphasizes that performance does not belong to a single discipline; it emerges from deliberate integration across training, medicine, psychology, and enabling technologies.

**Figure 5:**
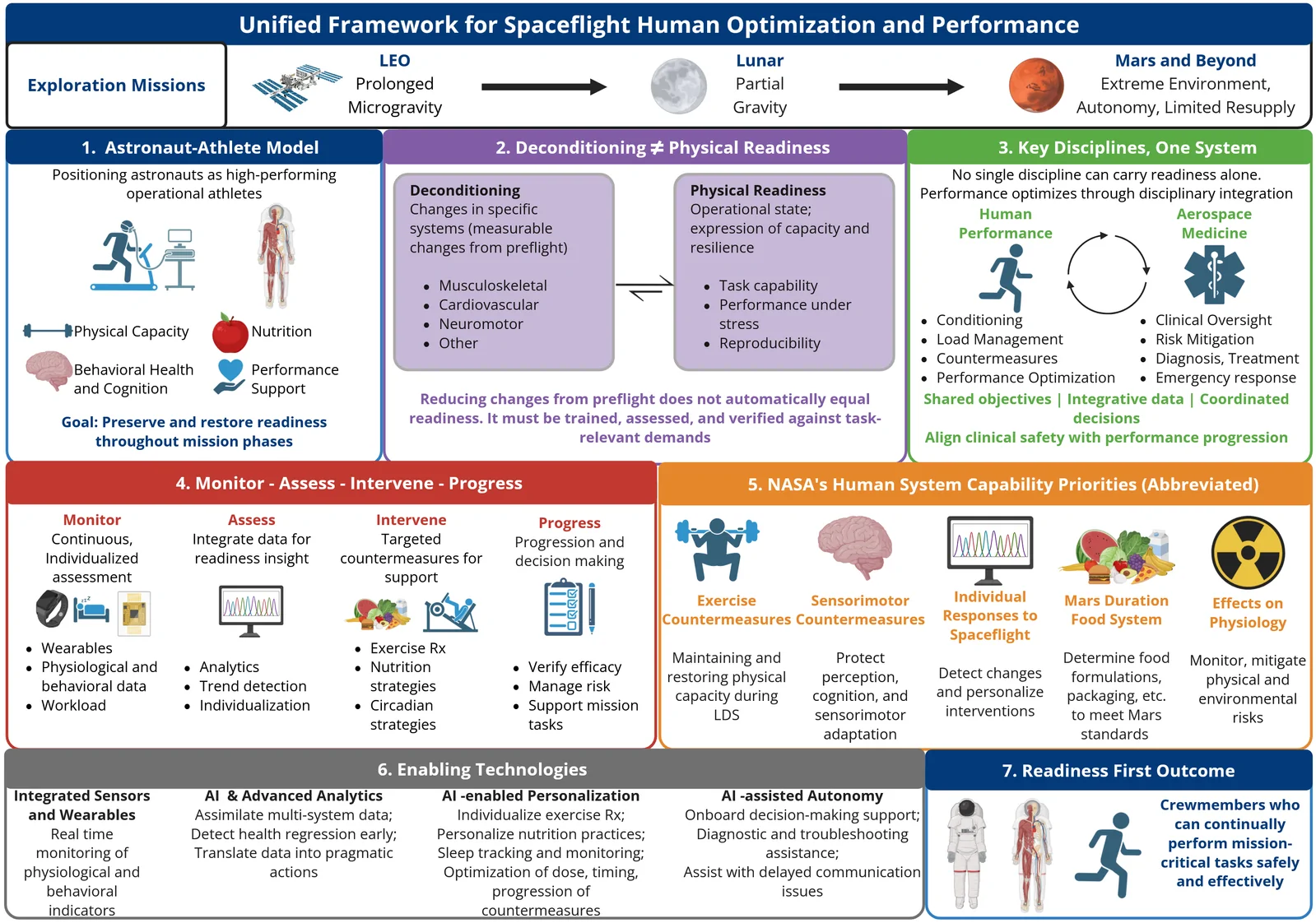
A unified framework for spaceflight human optimization and performance. This framework is proposed to situate astronauts within an athletic monitoring and progression model to optimally promote mission readiness, using a multidisciplinary approach that safely monitors and advances them through exercise training, behavior modification, and nutritional support, addressing the current human system capability needs prioritized for LDS.

A key clarification embedded in this perspective is that spaceflight-imposed deconditioning and physical readiness are related but not the same construct. Deconditioning reflects measurable shifts in specific systems, often characterized by changes from preflight baselines. Physical readiness, however, is an operational state—an integrated expression of capacity, resilience, movement quality, and tolerance to mission demands. Consequently, reducing the change between preflight and postflight metrics may indicate successful mitigation of certain physiological losses. Still, it does not automatically confirm that physical preparedness has been preserved or restored. Preparedness requires deliberate engagement: it must be trained, assessed, and verified against task-relevant demands, rather than inferred solely from narrower physiological markers. This distinction matters most for exploration missions, where performance must be repeatable under fatigue, stress, and constrained resources—conditions that can expose gaps between “recovered numbers” and true operational readiness. An interdisciplinary approach is integral in this regard, bridging aerospace medicine, human performance, nutrition, enabling technologies (wearables, AI, VR), and engineering that promote optimizing and/or restoring operational readiness for mission demands.
